# AI Caretaker: An Intelligent Voice-Activated Support System for Dementia Care

**DOI:** 10.7759/cureus.115149

**Published:** 2026-08-25

**Authors:** Aryan Dhingra, Charissa M Nichols

**Affiliations:** 1 Behavioral Health, Christian Health Care Center, Wyckoff, USA; 2 Psychiatry, Bergen New Bridge Medical Center, Paramus, USA; 3 Psychiatry/Addiction, Pine Rest/Michigan State University College of Human Medicine, Grand Rapids, USA

**Keywords:** artificial intelligence, assistive technology, dementia care, natural language processing, python, voice recognition

## Abstract

Introduction

Dementia care frequently requires continuous supervision, a demand that becomes difficult to meet when caregivers must balance outside responsibilities, particularly in home-based care, where unsupervised periods expose dementia patients to wandering, missed medication, loneliness, and a multitude of injuries. Existing tools often depend on manual patient input, an unrealistic expectation for individuals with impaired memory and judgment. This study presents the AI Caretaker, a voice-interactive support system that assists patients through natural conversation rather than commands or visual interfaces, and evaluates its technical feasibility.

Methods

Implemented as a tabletop device, the system continuously listens, interprets patient speech, classifies it as daily activity, medication intake, emergency, or casual conversation, logs detections to a relational database, and, when an emergency is identified, assigns a severity score and alerts the caregiver. The system was evaluated using 12 scripted scenarios spanning clear emergencies, nonemergency controls, ambiguous statements, and extended multiturn interactions, each executed across 10 trials, yielding 120 total trials and 440 turn-level statements processed through the complete pipeline.

Results

Speech recognition achieved a mean word accuracy of 94.6% (SD 15.8 percentage points). Binary emergency classification in clear scenarios was correct in 46 of 60 trials (76.7%) under a strict criterion requiring a correct classification on every statement (sensitivity: 26 of 30 trials, 86.7%; specificity: 20 of 30 trials, 66.7%), although every clear emergency trial generated at least one emergency flag. All 10 false-positive trials arose from emotional-distress statements and received uniformly low severity scores (median 10 of 100), indicating that the binary emergency flag and the continuous severity score were not calibrated with one another. Mean severity scores differentiated the three clear emergency types (79.0, 50.8, and 34.8 for fall with injury, progressive medical distress, and nighttime confusion, respectively). However, severity did not accumulate consistently across multiturn conversations, as the AI isolated the most recent statement rather than interpreting the dialogue as a whole. Mean end-to-end latency was 3.24 seconds against a 2.0-second target.

Conclusions

These findings support the feasibility of passive conversational monitoring in home-based dementia care and identify the flag-severity calibration needed to limit alert-fatigue risk, together with the temporal reasoning and latency improvements required before real-world deployment.

## Introduction

Problem

Dementia patients and their family caregivers face challenges, as caregivers attempt to balance employment and other responsibilities with providing consistent care. Unlike institutionalized patients, individuals living at home often experience extended periods of being alone or under limited supervision, creating stress for both the patient and the caregiver [1]. Caregivers continuously worry about medication adherence, daily activities, and home emergencies, often monitoring cameras or checking in remotely during work hours. At the same time, dementia patients experience feelings of loneliness and inadequacy as impairments in memory, task tracking, and judgment limit their ability to carry out activities of daily living or to communicate with others while alone [2]. These challenges are most prevalent in families supporting individuals with mild dementia, typically adults in their 60s through 80s, who remain verbally articulate yet experience cognitive impairments that can compromise safety during extended unsupervised periods. Together, these factors reveal a critical gap in home-based dementia care that affects both patient well-being and caregiver effectiveness.

Demographic trends amplify the urgency of this problem. In the United States, approximately 7.2 million adults aged 65 and older currently live with Alzheimer’s disease, a number expected to rise as life expectancy increases, placing a growing strain on families serving as primary caregivers [3]. Globally, dementia prevalence is projected to increase sharply in the coming decades as populations age, intensifying demand for scalable, in-home care solutions [4,5]. Beyond routine caregiving challenges, dementia presents serious safety risks. Research indicates that a substantial proportion of individuals with Alzheimer’s disease, by some estimates, a majority, exhibit wandering behavior at some point. However, reported prevalence varies considerably by care setting and dementia severity [6,7]. Wandering increases the likelihood of going missing, exposure to dangerous environments, or unsafe interactions with strangers. These incidents may go unnoticed for extended periods, creating gaps in supervision that can escalate into safety crises.

Current technology

Several state-of-the-art approaches are currently used to support in-home dementia patients and reduce caregiver risk. One standard solution is personal emergency response systems, such as wearable emergency call buttons offered by services like Life Alert [8]. These systems depend on the patient recognizing an emergency and pressing a button, an assumption that is often unrealistic for individuals with cognitive impairment. In situations involving confusion or panic, patients may forget to activate the device or be unable to do so [9,10]. Another widely used approach involves global positioning system (GPS) tracking and geofencing devices designed to address wandering behavior [11]. While these tools can alert caregivers after a patient leaves a predefined area, they offer limited insight into emergencies that occur within the home. Voice-based consumer technologies, such as smart speakers, reduce physical effort by relying on speech but still impose interaction barriers through required wake words and structured commands [12]. Overall, existing solutions either impose excessive cognitive demands on dementia patients or fail to provide passive activity logging and reliable emergency detection, exhibiting a persistent gap in current in-home dementia care technologies.

Solution

The solution is the AI Caretaker, a device that provides safety and monitoring within the home and can be used with patients who have mild dementia. This solution consists solely of voice-activated interaction to minimize patient effort. Audio input from the patient is collected via a microphone and interpreted using speech recognition. This text is then evaluated using AI, which systematically assesses it to identify emergencies, medication adherence, or activities. When an emergency is recognized, the system sends a text message to the caregiver containing the recognized message and the AI’s inferred severity level so that the caregiver can make decisions. After analysis, the AI facilitates dialogue, helping to reduce the loneliness and emotional distress a patient may experience when the caregiver is absent [13]. All conversations and detections are recorded in a structured query language (SQL) database and accessed via a caregiver-facing application. This method addresses the essential needs of caregiving by allowing the logging of passive activity and the detection of an emergency in real time, without the patient having to interact with devices, buttons, or applications.

In contrast to current solutions, the AI Caretaker combines the strengths of multiple existing tools to address their individual limitations. Wearable emergency-button devices notify caregivers only after a button is manually pressed and do not offer any conversation or record-keeping. GPS tracking systems only detect when the wanderer has actually changed location and do not provide information about emergencies within the home or about everyday activities. Consumer voice solutions like Amazon Alexa operate only after hearing wake words and do not perform activity recording or emergency inference [12]. Through entirely server-side AI analysis, the AI Caretaker enhances current solutions by reducing the patient’s input to speech alone while combining communication, record-keeping, and emergency awareness into a single backend platform.

## Materials and methods

System overview

The AI Caretaker was developed as a context-aware conversational monitoring system intended to support individuals with dementia who remain in home environments for extended periods without continuous direct supervision. The system was designed to combine natural voice interaction with automated analysis of safety-related events, medication behavior, daily activities, and changes in cognitive or emotional state. Rather than requiring the patient to activate a panic button, open an application, or provide information through a structured interface, the system accepts spontaneous spoken input. It processes it using a distributed, hardware- and application programming interface (API)-based, cloud-resident software architecture.

The implemented system consisted of four principal components: a local patient-facing voice device, a cloud-hosted inference backend, a relational database, and a caregiver-facing web dashboard (Appendix A). The local device captured patient speech and delivered synthesized responses. The backend processed speech transcripts, retrieved recent conversational history, invoked a large language model (LLM), generated a supportive response, and classified clinically relevant information. The database stored patient, caregiver, conversational, medication, activity, emergency, and severity data. The dashboard presented these records remotely to an authorized caregiver.

Figure 1 presents the overall architecture of the system and the integration and data flows among its four components. Patient speech was captured by the local device and converted into text before being transmitted to the backend. The backend retrieved recent conversation history from the database, analyzed the transcript, generated a structured response, and returned conversational text for speech playback. Information identified during the analysis was simultaneously recorded in the database and made available through the caregiver dashboard. When an emergency was detected, the backend initiated an alerting workflow using caregiver contact information stored in the database.

**Figure 1:**
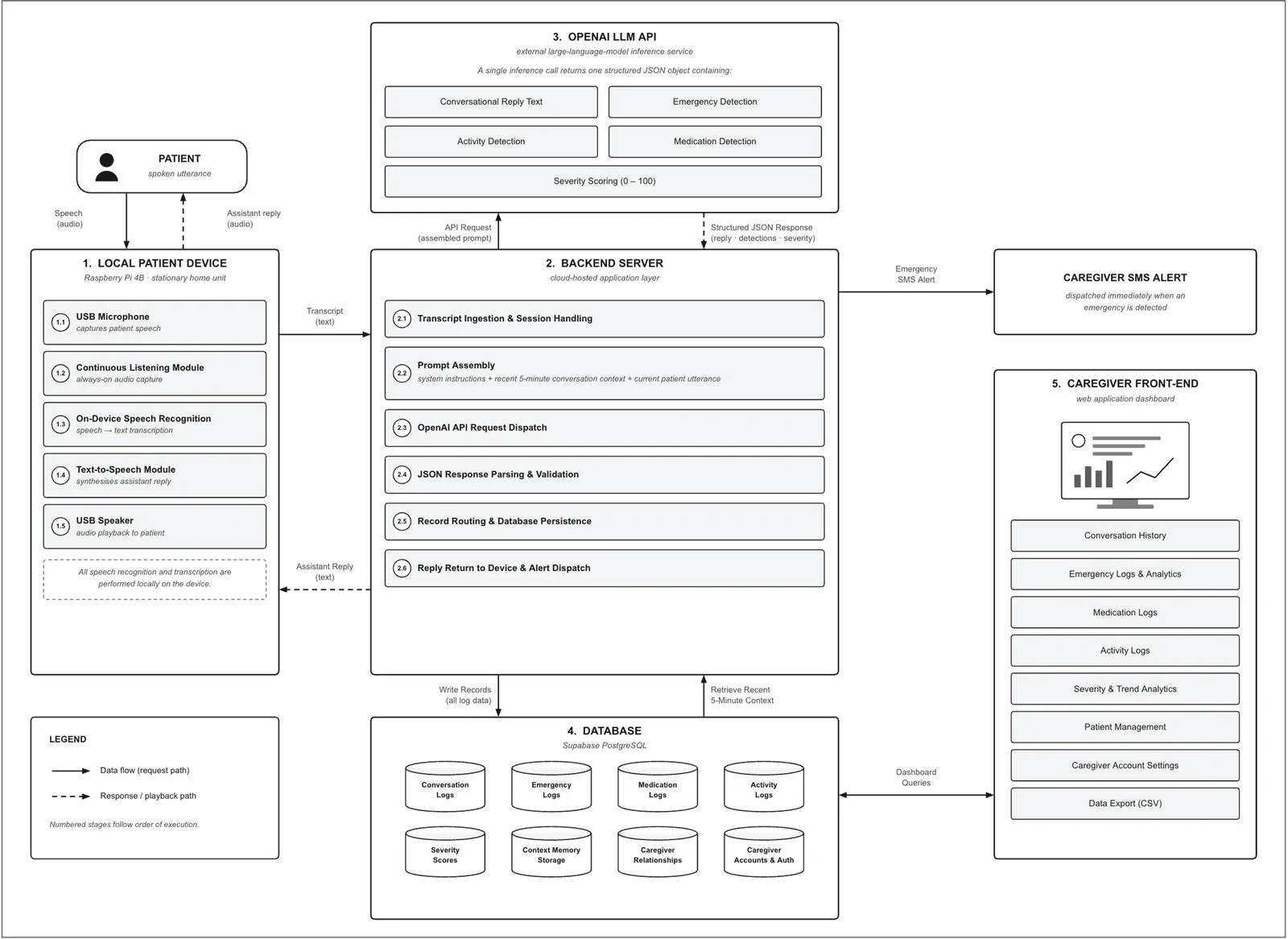
System architecture of the AI Caretaker dementia monitoring system showing the local patient device, cloud backend, database, and caregiver dashboard. API: application programming interface; CSV: comma-separated values; JSON: JavaScript Object Notation; LLM: large language model; SMS: short message service; USB: universal serial bus This figure was created using Microsoft PowerPoint (Microsoft Corporation, Redmond, WA, USA).

The prototype was designed as a distributed system rather than a fully local application because different tasks required different computational resources, as well as for scalability and parallel processing. Audio capture and playback were performed by the patient-facing device, while language-model inference, contextual analysis, classification, and notification logic were managed by the backend. This separation allowed the local hardware to remain compact and relatively inexpensive while enabling more computationally intensive processing through cloud-hosted services. The database and dashboard were maintained as separate components so that conversational data could be retained longitudinally and reviewed independently of the local device.

Local voice interaction device

The local patient-facing device was built using a Raspberry Pi 4B connected to a universal serial bus (USB) microphone and USB audio-output hardware. The Raspberry Pi provided wireless network connectivity, local audio capture, speech-recognition integration, and playback of generated responses. The evaluated prototype was designed as a stationary tabletop unit for placement in a commonly occupied area of the patient’s residence. Although only one unit was tested, the architecture could support additional room-based devices linked to the same patient account, backend, and database as seen in Figure 2. This would allow interactions captured in different rooms to contribute to a shared conversational history. Such a multidevice configuration was not evaluated in the present study.

**Figure 2:**
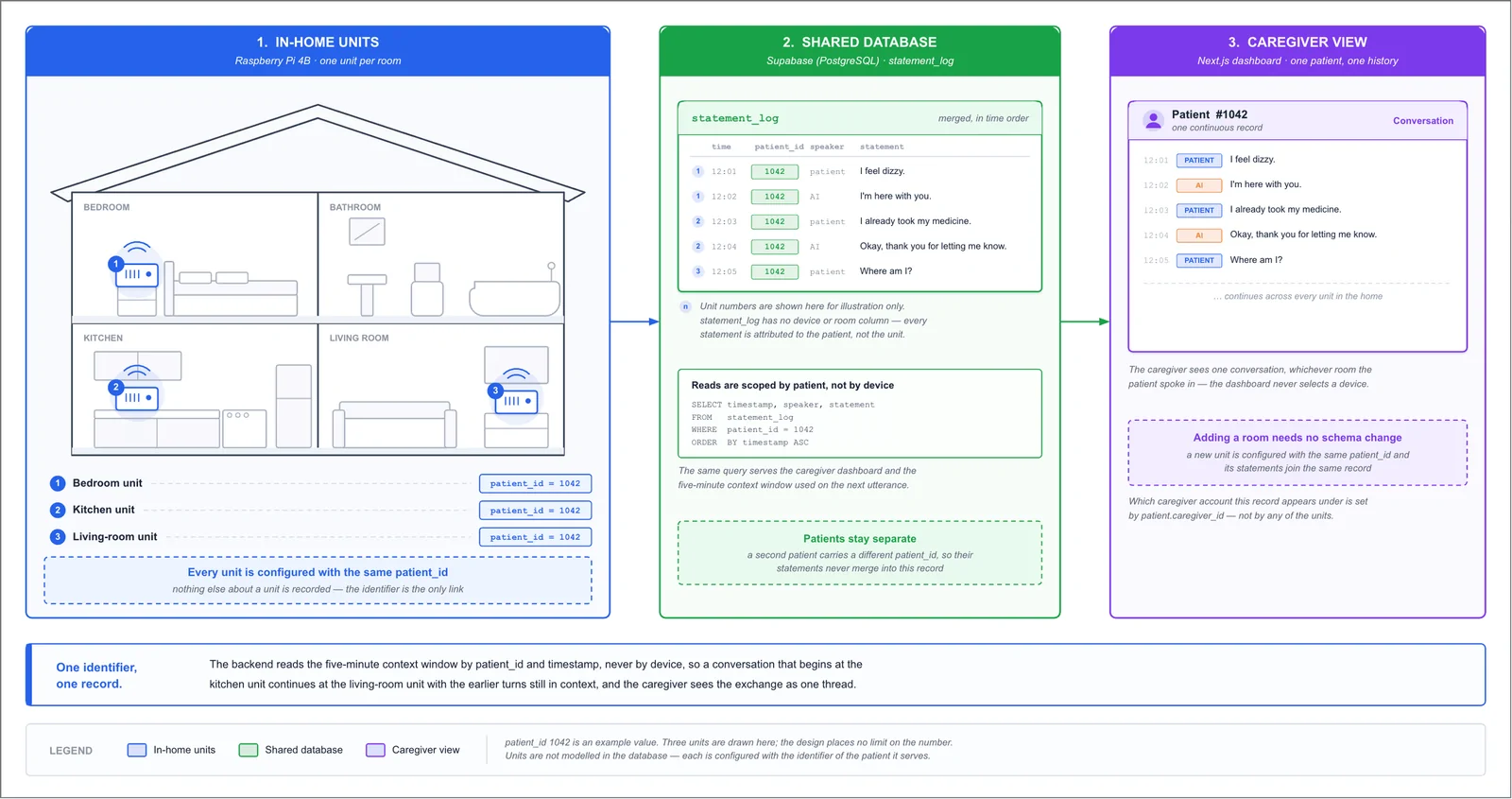
Multiroom deployment linked by a shared patient identifier. Every unit in the home reports under the same patient_id, so all statements merge into one record and one caregiver view. This figure was created using Microsoft PowerPoint (Microsoft Corporation, Redmond, WA, USA).

Transcription was performed on the device using the open-source Python SpeechRecognition library configured with its default recognizer, the free Google Web Speech API (the recognize_google function) [14]. The energy threshold defined the minimum sound level required for the system to treat incoming audio as speech. This value was selected through repeated testing to identify a setting that reliably detected the speaker’s voice while limiting activation from ambient room noise. The pause threshold determined how long the system waited during silence before considering an utterance complete. A slightly longer pause threshold was selected to accommodate the hesitations and extended pauses that may occur in the speech of individuals with dementia, reducing the likelihood that a statement would be divided or cut off prematurely [15].

After an utterance was detected, the captured audio was passed to the speech-recognition component and converted into text. The transcript was then transmitted to the backend for contextual analysis and classification. Once processing was completed, the backend returned a natural-language response, which was converted into synthesized speech and played through the device’s speakers. This created a closed conversational loop in which spoken input produced both an audible response and a structured safety assessment.

The local device did not independently determine whether a statement represented an emergency. Its primary responsibilities were speech acquisition, utterance detection, transcript generation, network communication, and audio playback. Emergency detection, contextual-memory retrieval, severity scoring, database logging, and caregiver notification were performed by the centralized backend.

Speech recognition and audio processing

Patient speech was converted into machine-readable text using a speech-to-text pipeline integrated with the Raspberry Pi. Before transcription, the system calibrated the microphone input and monitored ambient sound to establish an appropriate energy threshold. Audio was segmented into phrases based on detected speech activity and silence intervals. This segmentation prevented prolonged background audio from being submitted as a single transcript and allowed individual patient statements to be processed as conversational turns.

The speech-recognition pipeline was configured to support several characteristics relevant to dementia care, including low-volume speech, incomplete sentences, delayed responses, repeated phrases, and emotionally distressed speech features that are known to challenge automatic speech recognition in older and cognitively impaired speakers [16]. Each recognized phrase was converted into a transcript that preserved the patient’s spoken wording as closely as possible. The transcript was then sent to the backend together with the patient identifier required for contextual retrieval and database association.

Speech recognition was evaluated within the complete AI Caretaker workflow because transcription quality directly influenced every later stage of processing. During each scripted trial, the spoken statement was transcribed by the system, and the generated transcript was compared with the original script at the word level. Errors were categorized as omissions, substitutions, or complete recognition failures. This approach allowed evaluation of not only overall transcription accuracy but also whether clinically important words, such as terms describing pain, falls, dizziness, confusion, or medication intake, were preserved. Because the transcript served as the input for emergency classification, severity scoring, activity detection, medication logging, and response generation, even a small transcription error could alter the system’s final interpretation. Speech-to-text performance was therefore measured as part of the end-to-end scenario evaluation rather than as a separate laboratory test.

Conversational processing and inference pipeline

The cloud-hosted backend functioned as the central processing layer of the system. It received the current patient transcript, retrieved recent conversational records, assembled the model input, invoked an LLM, validated the returned structured output, stored the resulting data, and returned a conversational reply to the local device.

Figure 3 illustrates this end-to-end processing sequence. The pipeline began when the local device transmitted a patient transcript. The backend then queried the database for recent statements associated with that patient. These statements included both patient speech and prior AI responses so that the model could interpret the current statement within the context of the ongoing exchange. The current transcript and retrieved history were combined with system instructions describing the required response format and safety-classification tasks.

**Figure 3:**
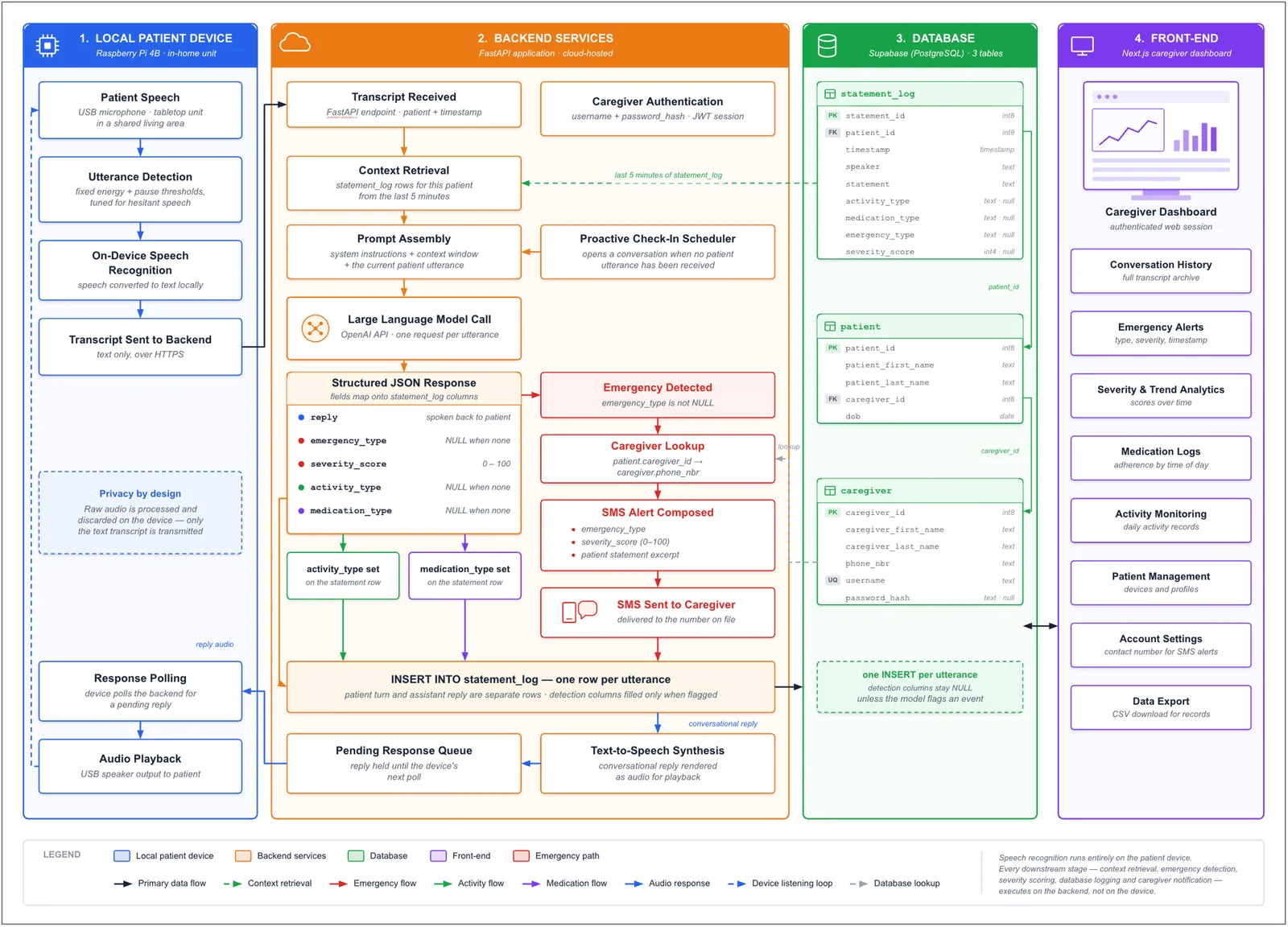
Conversational processing pipeline of the AI Caretaker dementia monitoring system, from patient speech to structured output, database logging, and caregiver notification. API: application programming interface; HTTPS: hypertext transfer protocol secure; JSON: JavaScript Object Notation; LLM: large language model; SMS: short message service; SQL: structured query language; USB: universal serial bus This figure was created using Microsoft PowerPoint (Microsoft Corporation, Redmond, WA, USA).

Language-model inference was performed with OpenAI’s GPT-4o mini model (gpt-4o-mini; OpenAI, San Francisco, CA, USA), accessed through the OpenAI API using default generation parameters; no custom temperature or sampling settings were applied. GPT-4o mini was selected because the inference task in this system involves generating a brief supportive reply alongside a fixed JavaScript Object Notation (JSON) structure containing Boolean flags, categorical fields, and a bounded severity score. This represents a constrained classification and structured-output problem rather than open-ended clinical reasoning, and low per-request latency was essential to sustaining a natural conversational loop. This tradeoff is supported by recent clinical natural-language-processing work: in a controlled evaluation of extraction strategies on electronic health record discharge summaries, GPT-4o mini achieved accuracy competitive with larger and fine-tuned alternatives at the lowest token cost, indicating that lightweight instruction-tuned models are adequate for bounded clinical extraction and classification tasks at a fraction of the computational expense of frontier models [17].

Several algorithmic properties of the model underlie inference in this application. GPT-4o mini is a decoder-only transformer that generates output autoregressively, predicting one token at a time while computing multihead self-attention over the entire assembled prompt [18]. Self-attention is the mechanism through which the retrieved five-minute conversational window is integrated into each prediction: every generated token can attend to, and weight, all preceding context tokens, so the emergency, activity, and medication judgments are conditioned on the accumulated dialogue rather than on the current utterance in isolation. Because the attention keys and values computed for earlier tokens are cached and reused at each decoding step rather than recomputed, response generation remains fast enough for near-real-time interaction [18]. Finally, the model’s adherence to the strict JSON output structure from a minimal system instruction, without task-specific fine-tuning, derives from instruction tuning with reinforcement learning from human feedback, which aligns generated outputs with user-supplied instructions and formatting constraints [19].

Because sampling remained at default values, model outputs were nondeterministic, and repeated presentations of an identical script could yield different classifications, the mechanism underlying the run-to-run variability observed across trials. Each request combined a fixed system prompt, the retrieved conversational history, and the current transcript. The system prompt was deliberately minimal: it assigned the model the role of a caregiver assistant for a dementia patient, directed the reply to remain calm, simple, and safe, and constrained the output to a strict JSON structure containing a conversational reply and three detection objects. The emergency object comprised a Boolean flag, a free-text type, and a severity score from 0 to 100; the activity object comprised a Boolean flag and a free-text type; and the medication object comprised a Boolean flag and a time-of-day field (morning, evening, or unknown). No explicit decision rules, category definitions, or scoring rubric were provided, so classification and severity judgments relied entirely on the model’s own reasoning over the assembled context. This structure allowed the conversational reply to be separated from the machine-readable classifications used by the database, notification system, and dashboard.

The system prompt was as follows:

You are a caregiver assistant for a dementia patient.

Be calm, simple, and safe.

Return ONLY JSON:

{

 "reply": "...",

 "detections": {

 "emergency": {"flag": true/false, "type": "string", "score": 0-100},

 "activity": {"flag": true/false, "type": "string"},

 "medicine": {"flag": true/false, "time_of_day": "Morning/Evening/Unknown"}

 }

}

Because the emergency and activity type fields were free text rather than a predefined taxonomy, the model generated its own labels. Types observed during testing included falls, chest-related distress, dizziness, confusion, and loneliness-related distress for emergencies, and eating, walking, and social interaction for activities. Medication detections recorded whether a medication event was mentioned and, when stated, the associated time of day.

A single patient statement could produce more than one classification. For example, a statement describing dizziness while walking could be logged as both an activity-related event and a potential emergency. Similarly, a patient who reported taking the wrong medication could generate a medication record and an emergency classification. This multilabel approach was selected because real-world patient speech does not necessarily conform to one isolated clinical category.

The system prompt directed the generated reply to remain calm, simple, and safe. In practice, during potential emergencies, the generated replies encouraged the patient to remain in a safe position, continue communicating, or wait for caregiver assistance, while in nonemergency situations the replies provided companionship, acknowledged emotional concerns, or answered routine questions. The conversational response and the patient transcript were both stored so that later interactions could be interpreted in context.

Context-aware conversational memory

The system incorporated a rolling conversational-memory window to prevent each statement from being evaluated in isolation. For every new transcript, the backend retrieved approximately five minutes of recent conversation associated with the patient. Retrieved records were ordered chronologically and included timestamps, speaker labels, patient statements, and prior assistant responses.

Figure 4 shows the process used to assemble this context before LLM invocation. The backend first queried the Supabase database for recent conversation records. These entries were compiled into a formatted pre-prompt representing the preceding interaction. The current patient transcript was then appended as the most recent input. The combined prompt was submitted to the model for contextual inference and response generation. After processing, the new patient statement and assistant response were written back to the database, allowing the conversational memory to update continuously.

**Figure 4:**
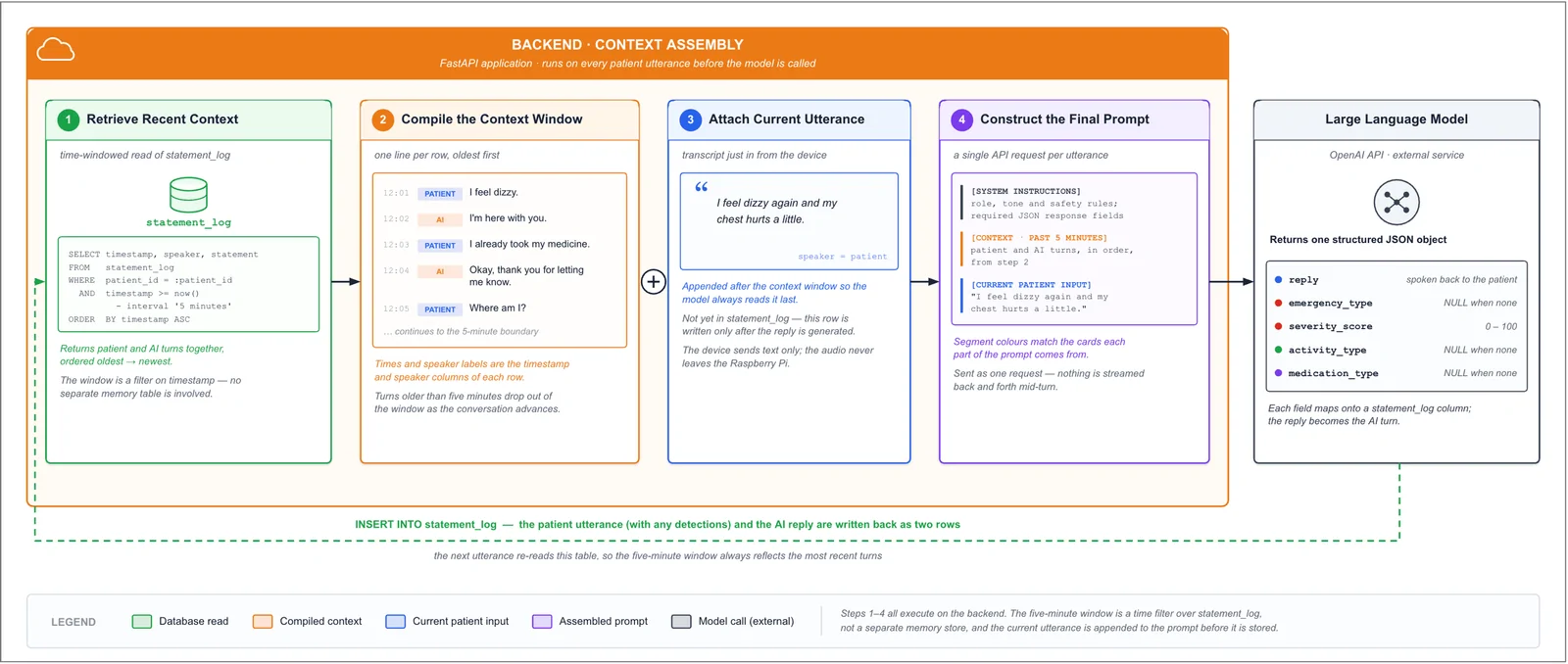
Backend context assembly prior to LLM invocation showing retrieval of the recent five-minute window, pre-prompt construction, and continuous memory update. API: application programming interface; JSON: JavaScript Object Notation; LLM: large language model; SQL: structured query language This figure was created using Microsoft PowerPoint (Microsoft Corporation, Redmond, WA, USA).

The five-minute context window was selected to preserve immediate conversational continuity while limiting the amount of unrelated historical information included in each request. This approach allowed the system to identify progression across several statements. A phrase such as “I feel strange” might not independently justify a high emergency score. However, if followed by “I feel dizzy,” “I need to sit down,” and “I can’t stand,” the combined history provides evidence of an escalating event. The same contextual mechanism also supports resolution of contradictory medication statements, repeated confusion, and changes from emotional distress to disorientation.

Conversational memory was therefore used not merely to improve dialogue quality but also as an input to risk classification. Emergency status and severity could change as new statements were added, even when no single statement contained an explicit emergency keyword.

Emergency classification and severity scoring

For each processed statement, the model returned a Boolean emergency flag and, when the flag was positive, a free-text emergency type and a severity score from 0 to 100 representing the estimated urgency of the event given the current statement and the retrieved conversational context. When no emergency was flagged, no severity value was produced; for all analyses in this study, such statements were assigned a severity of 0. Because the prompt supplied no scoring rubric, the severity value reflected the model’s own judgment of urgency.

Scores from 0 to 30 were interpreted as low-risk or nonemergency events, scores from 31 to 69 represented uncertain or moderate-risk situations, and scores from 70 to 100 indicated high-risk events that could require caregiver escalation. These ranges were used as operational categories rather than clinically validated diagnostic thresholds. The system was not designed to replace medical judgment, emergency services, or direct caregiver assessment.

The scoring process allowed risk to change over time. In an escalating conversation, an initially low or moderate score could increase as additional symptoms were reported. Conversely, clarifying statements could reduce apparent risk. For example, figurative language such as “That movie almost killed me” should not remain classified as an emergency after the patient clarified that the statement referred to an intense film and that they were physically well.

Caregiver notification workflow

When the model returned a positive emergency flag, the backend initiated the caregiver-notification pathway. The binary flag alone determined whether an alert was generated; the severity score did not modify the trigger condition. The patient record identified the associated caregiver, whose stored contact information was used to compose an alert containing the patient’s name, the detected emergency type, the severity score, the relevant patient statement, and the event timestamp, formatted for transmission as a text message through Verizon’s carrier email-to-short message service (SMS) gateway using the Simple Mail Transfer Protocol, an approach selected to avoid per-message messaging-service costs during prototyping. The workflow operated passively and did not require the patient to request help explicitly, a central design consideration because an individual experiencing confusion, injury, or cognitive impairment may not remember how to activate a conventional emergency device. Because alert dispatch is a deterministic code path executed whenever the flag is returned, message transmission was not exercised during the scripted trials; the evaluation assessed the detection and classification stages that determine whether an alert is generated, and alert composition was verified functionally outside the trial framework, as seen in Figure 5.

**Figure 5:**
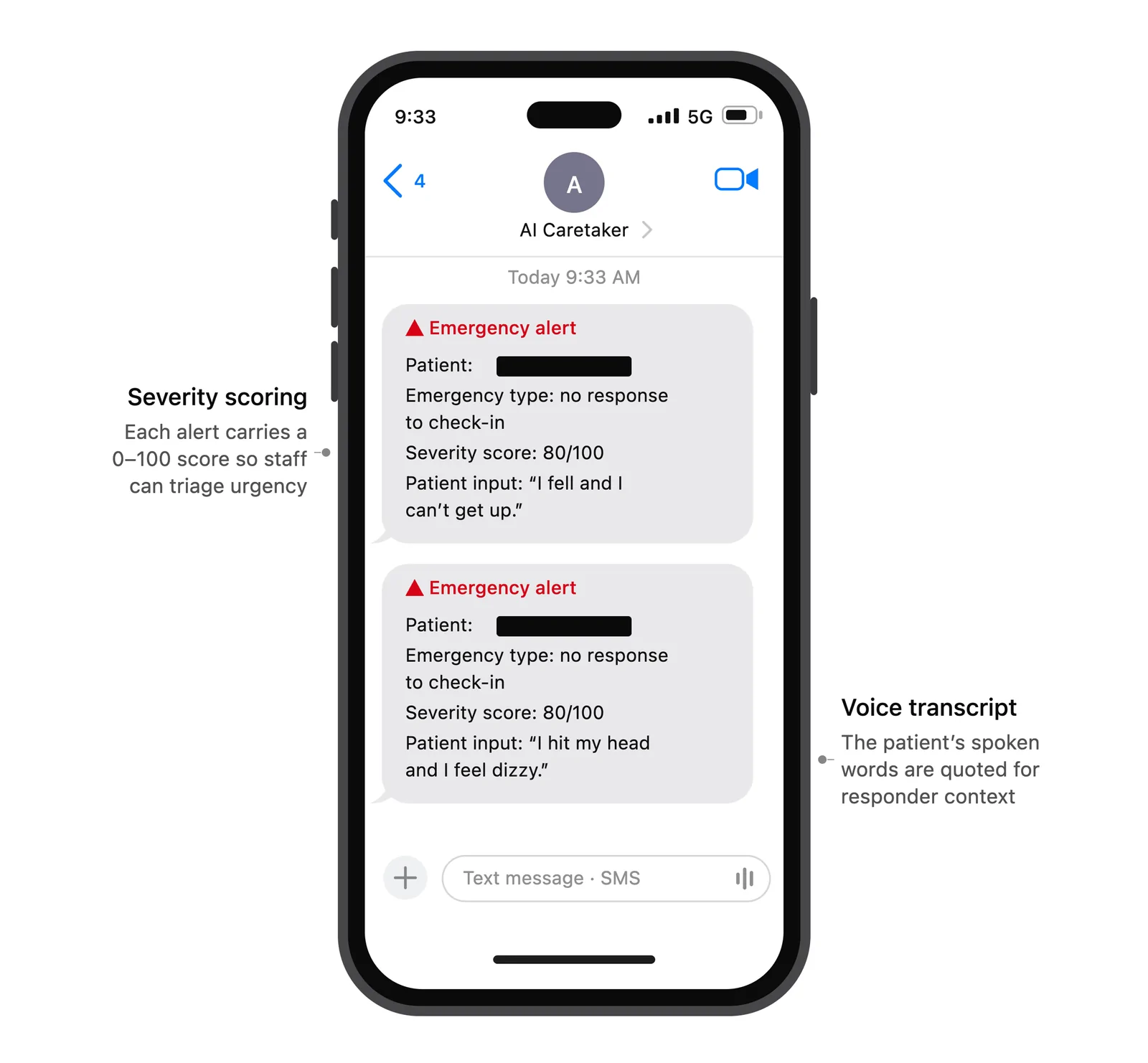
AI Caretaker’s automated SMS emergency alerts showing the AI-assigned severity score and the patient’s transcribed speech. SMS: short message service This figure was created using Microsoft PowerPoint (Microsoft Corporation, Redmond, WA, USA).

All identified events remained available in the database regardless of whether a notification was transmitted. This allowed lower-severity events, repeated moderate-risk statements, and false-positive classifications to be reviewed during evaluation.

Database architecture

A Supabase-hosted PostgreSQL database was used to store patient, caregiver, conversational, and classification data. The implemented relational structure is shown in Figure 6. The caregiver table stored a caregiver identifier, first and last names, and contact number. The patient table stored a patient identifier, name, date of birth, and a caregiver foreign key. This relationship allowed one caregiver to be associated with multiple patients while linking each patient to the caregiver responsible for receiving alerts and reviewing records.

**Figure 6:**
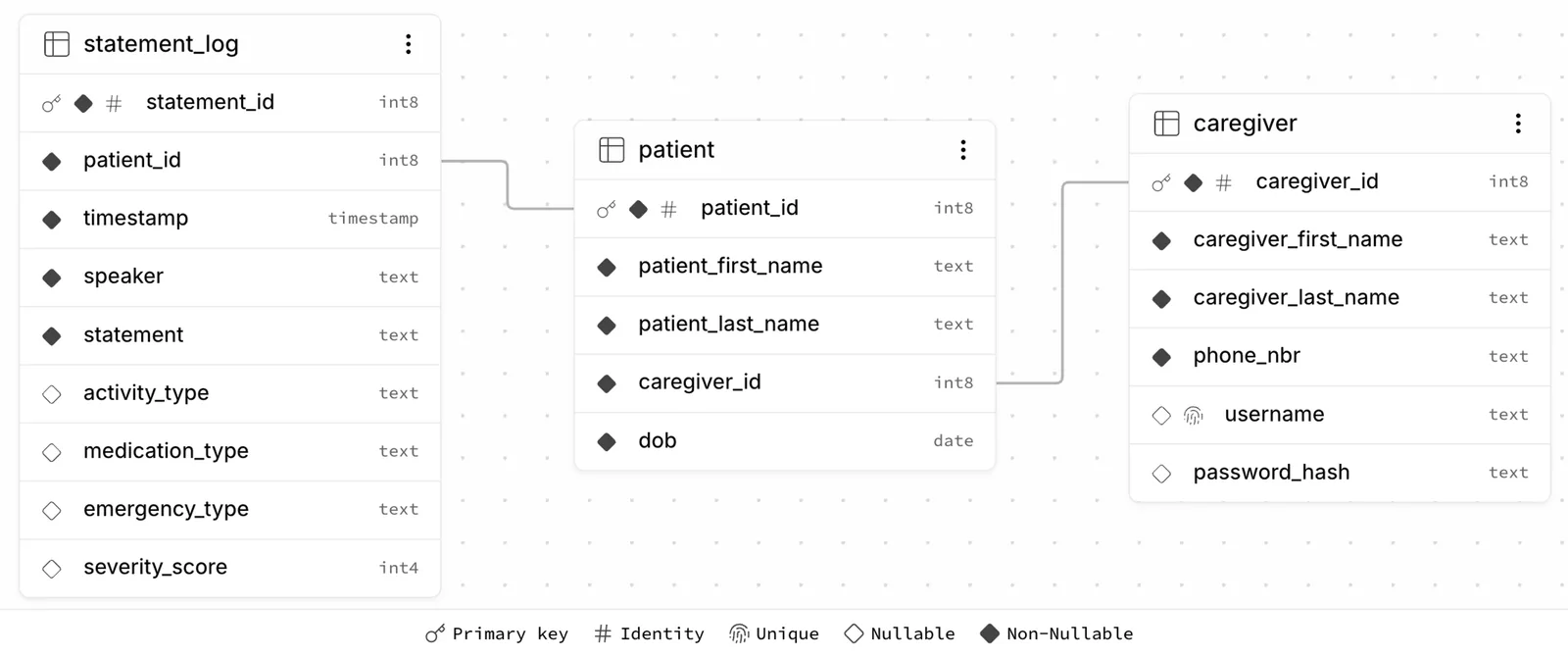
Supabase SQL database schema of the AI Caretaker dementia monitoring system showing the caregiver, patient, and statement-log tables and their relationships. SQL: structured query language

The statement-log table stored each patient or assistant utterance as a separate timestamped record. Fields included the statement identifier, patient identifier, timestamp, speaker, conversational text, activity type, medication type, emergency type, and severity score. Classification fields were nullable because most conversational statements did not contain all event categories. An emergency type and severity score were stored only when relevant, while routine statements could be retained without an emergency classification.

This structure supported both immediate processing and longitudinal review. During a conversation, the backend queried the statement log to retrieve recent context. For caregiver monitoring, the same records were filtered by patient, event type, severity, or timestamp. The database also allowed patient and assistant statements to be differentiated while preserving their chronological relationship.

Caregiver dashboard

A web-based caregiver dashboard was developed using a TypeScript frontend connected to the backend and Supabase database. The interface provided remote access to recent conversations, detected emergencies, medication events, activity records, timestamps, and severity information. The home page summarized the number of conversations, emergencies, medication entries, and activity events and displayed recent high-priority information.

Figure 7 presents the implemented dashboard interface. Recent patient and assistant statements were displayed in conversational order, while emergency events were shown separately with the detected type and severity score. Additional pages provided dedicated views for conversation history (Appendix B), emergency records, medication logs, and activity logs. The interface also included patient-management functions, caregiver account settings, and data-export capability.

**Figure 7:**
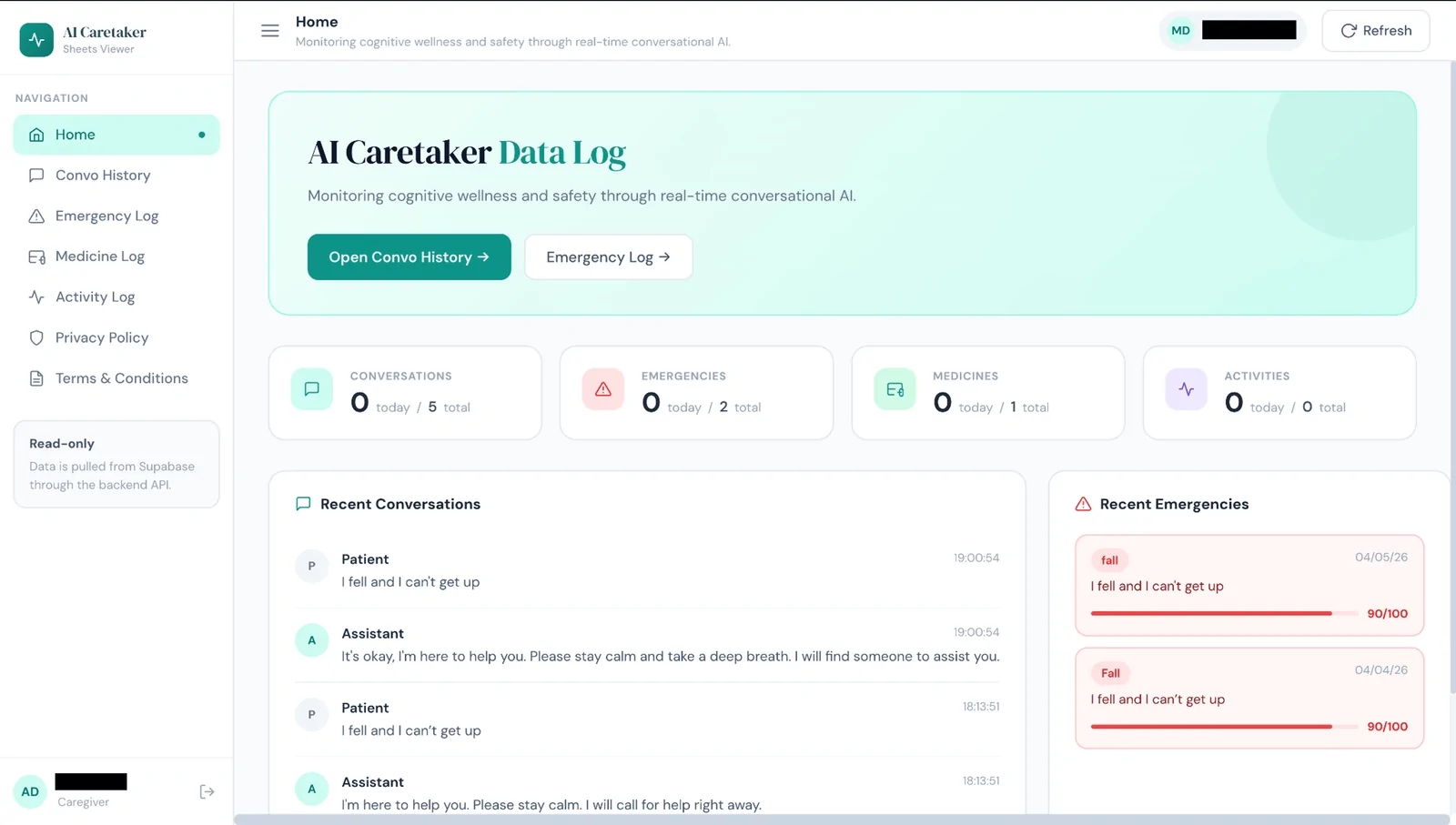
Screenshot of the home analytics page of the caregiver dashboard showing recent scripted data.

The dashboard was used during testing to confirm that outputs generated by the inference pipeline were stored correctly and could be retrieved through the frontend. It also provided a visual method for examining false positives, severity-score changes, medication inconsistencies, and emergency escalation across repeated trials.

Physical device construction

The patient-facing AI Caretaker was constructed as a compact, stationary tabletop unit housed within a custom enclosure. Interactive Model 1 presents the external casing, including the rectangular form factor, ventilated upper surface, and openings required for audio output and peripheral access.

**Video 1:** Interactive three-dimensional model of the AI Caretaker enclosure. The model presents the custom outer casing, including its tabletop form factor, ventilated upper panel, and openings for audio output and peripheral access.

The completed exterior assembly is shown in Figure 8. In this configuration, the internal electronics were enclosed within the casing, the two speakers projected audio through side-facing openings, and an externally positioned microphone captured speech from the surrounding environment.

**Figure 8:**
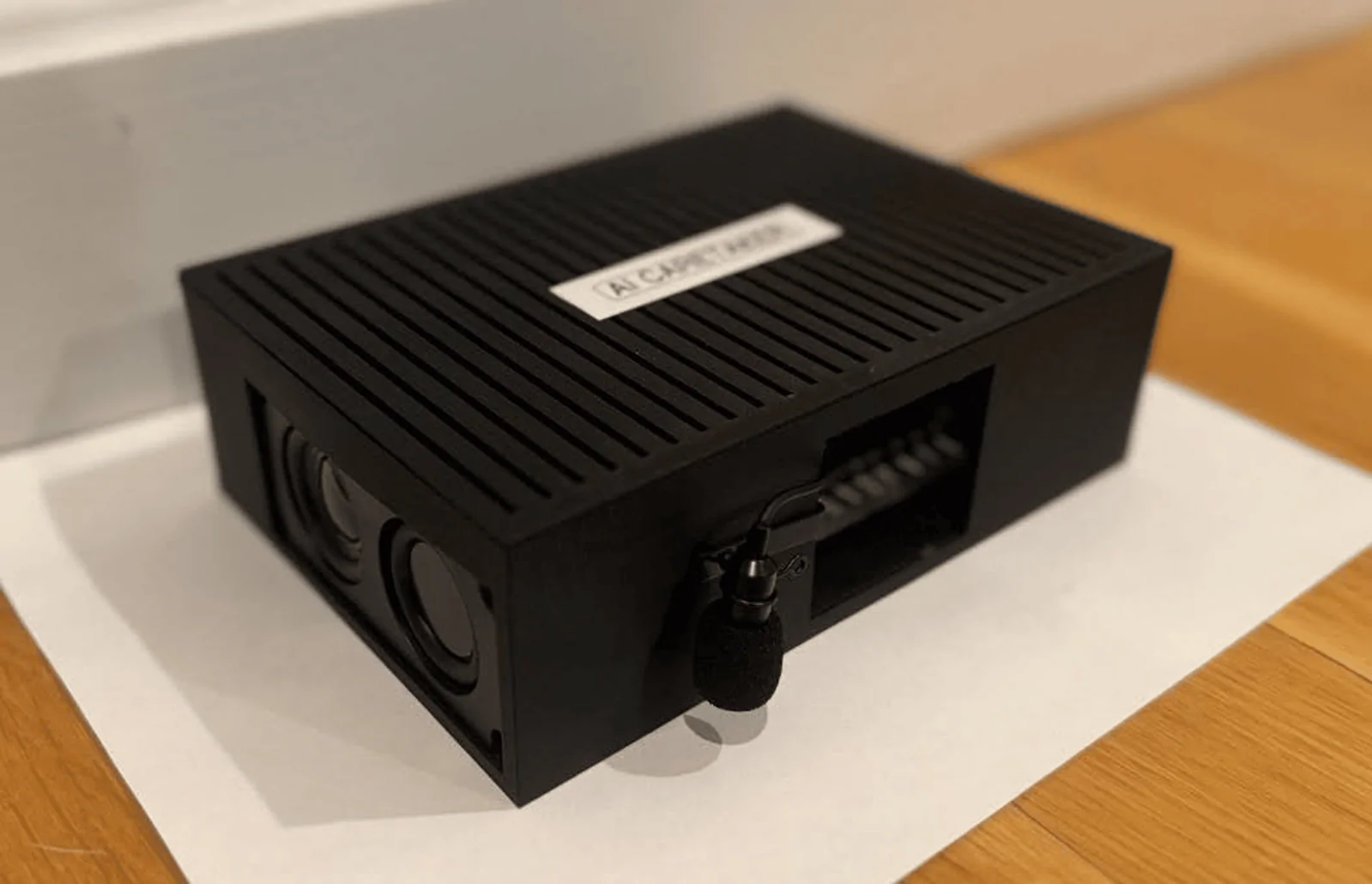
Exterior view of the fully assembled AI Caretaker prototype. The completed device is shown within its custom enclosure, with side-facing speaker openings and an externally positioned microphone for voice interaction.

The internal arrangement of the prototype is shown in Figure 9. A Raspberry Pi 4B served as the local computing platform and was mounted centrally within the enclosure alongside two speakers, a USB microphone, a speaker adapter, and the associated power and audio wiring. The components were arranged to create a self-contained device capable of capturing patient speech, communicating with the cloud-hosted backend, and reproducing synthesized responses without requiring the patient to operate a screen, button, or handheld controller.

**Figure 9:**
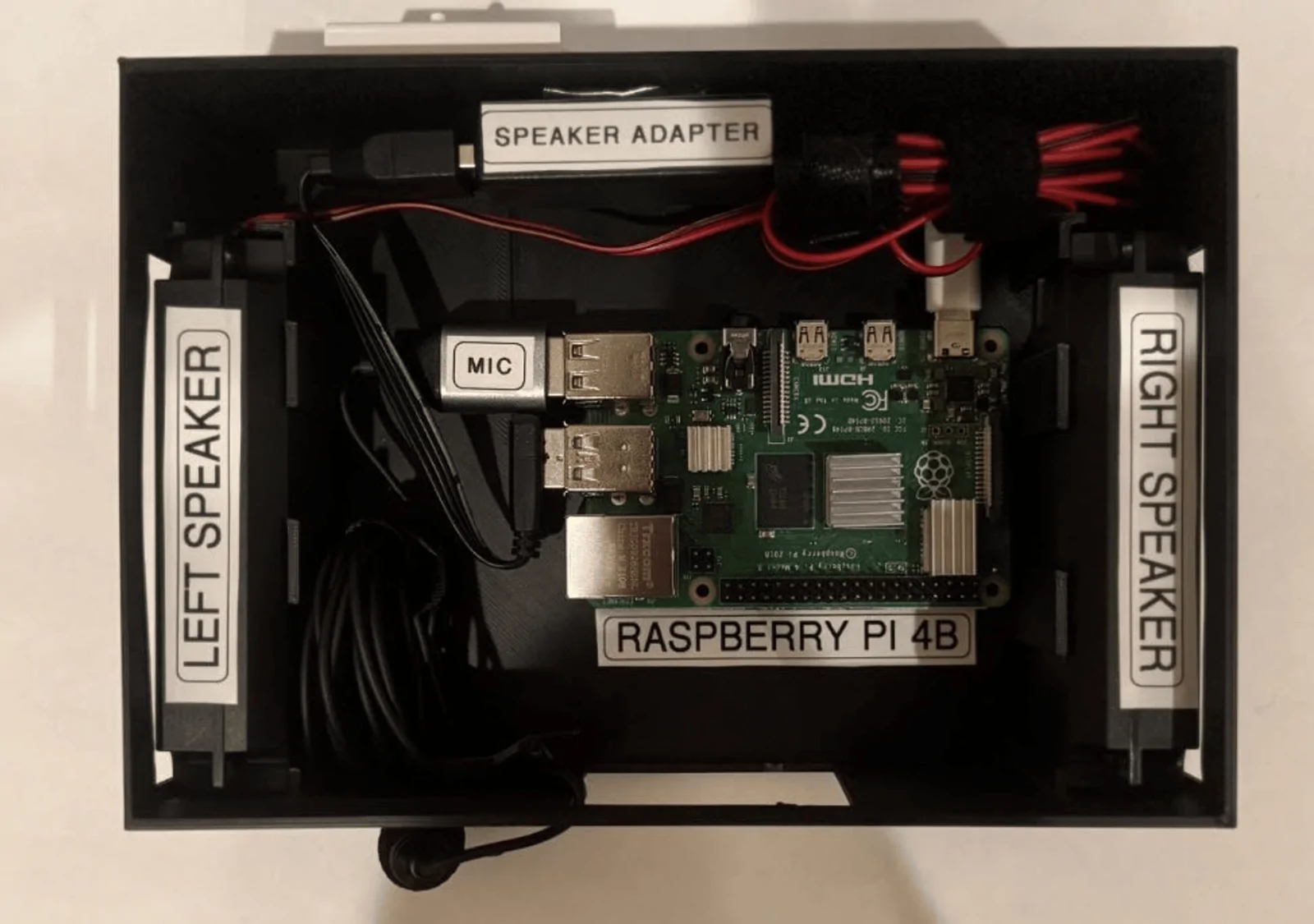
Internal component arrangement of the AI Caretaker prototype. The enclosure contains a centrally mounted Raspberry Pi 4B, two speakers, a USB microphone, a speaker adapter, and the associated power and audio wiring.

Study objectives

Six performance objectives were defined before testing began and are summarized in Table 1. Three specify numerical thresholds that the system either meets or fails; the remaining three state directional expectations about severity scoring, escalation behavior, and contextual-memory retention that were assessed qualitatively. Performance against all six objectives is reported in the Results.

**Table 1:** Prespecified performance objectives and their success criteria.

Objective no.	Domain	Objective	Success criterion	Criterion type
1	Speech recognition	Mean speech-to-text word accuracy across all transcribed statements.	≥90% mean word accuracy	Quantitative
2	Emergency classification	Binary emergency classification accuracy across the clear emergency and clear nonemergency scenarios.	≥90% classification accuracy	Quantitative
3	Severity scoring	Mean severity scores reflect the type and intensity of each clear emergency scenario.	Mean severity scores correspond to scenario type and intensity	Qualitative
4	Multiturn escalation	In the escalating multiturn scenario, severity increases consistently across successive statements.	Severity rises consistently across statements and culminates in an emergency classification	Qualitative
5	Contextual memory	The contextual-memory mechanism prevents a single reassuring statement from erasing previously accumulated risk, particularly in the conflicting-medication scenario.	Accumulated risk retained despite a reassuring statement	Qualitative
6	System latency	Mean end-to-end latency from patient utterance to system response.	≤2.0 seconds mean latency	Quantitative

Experimental design

The system was evaluated using a scripted scenario-based testing framework rather than through direct deployment with patients. The scenarios were developed a priori from two sources: informal consultations, conducted before testing, with geriatric behavioral health physicians regarding common, nonpatient-specific incidents and risks encountered by individuals with dementia living at home, and the event types documented in the cited literature, including falls [10], nighttime confusion [6,7], medication adherence, and loneliness [2,13]. These consultations informed the selection of event categories; the scripted statements themselves were authored by the investigators and were not independently reviewed or validated by clinicians. Because the system analyzes transcribed lexical content only, each event was constructed as an explicit verbal statement; the implications of this constraint for real-world performance are addressed in Study limitations.

The evaluation comprised 12 conversational scenarios divided evenly among four categories: clear emergencies, nonemergency controls, ambiguous edge cases, and extended multiturn interactions, each executed 10 times, yielding exactly 120 trials. Nine scenarios contained three scripted statements, and the three extended scenarios contained six, five, and six statements, producing 440 turn-level interactions in total. Each trial was processed through the full system pipeline, including voice input, speech recognition, backend transmission, contextual retrieval, LLM inference, structured output generation, database logging, and response playback. Table 2 presents each scenario’s scripted statements in order of testing and the expected behavior defined a priori during scenario design.

**Table 2:** Twelve scripted scenarios used in trials, with statement counts and a priori expected system behavior.

ID	Scenario	Category	Statements (n)	Scripted statements	Expected behavior (a priori)
A1	Fall with injury	Clear emergency	3	“Help, I fell.” / “I can’t get up.” / “My leg hurts a lot.”	Emergency flag on each statement; high severity (approximately 85-100)
A2	Medical distress escalation	Clear emergency	3	“I don’t feel good.” / “I feel dizzy.” / “My chest feels tight.”	Emergency flag; severity increasing across statements
A3	Severe nighttime confusion	Clear emergency	3	“I don’t know where I am.” / “It’s very dark.” / “I think I’m alone.”	Emergency or high-risk flag; severity approximately 70-90
B1	Routine daily interaction	Nonemergency control	3	“What time is it?” / “What’s the weather today?” / “Thank you.”	No emergency flag; any flag counted as a false positive
B2	Emotional but safe	Nonemergency control	3	“I feel lonely.” / “I miss my family.” / “Can you talk with me?”	Supportive response; no emergency flag; any flag counted as a false positive
B3	Metaphor and slang	Nonemergency control	3	“That movie almost killed me.” / “It was really intense.” / “I’m fine now.”	No emergency flag; any flag counted as a false positive
C1	Mild physical discomfort	Ambiguous	3	“My head hurts.” / “It’s probably nothing.” / “I just feel off.”	Indeterminate; moderate severity acceptable
C2	Hesitant disclosure	Ambiguous	3	“I don’t want to bother anyone.” / “Something feels strange.” / “I think I’m okay though.”	Indeterminate; variability expected
C3	Cognitive drift	Ambiguous	3	“What day is it?” / “That doesn’t sound right.” / “I’m confused.”	Borderline; moderate severity plausible
D1	Escalating ambiguity	Extended multiturn	6	“I feel strange.” / “My head hurts a little.” / “I’m probably fine.” / “I feel dizzy.” / “I need to sit down.” / “I can’t stand.”	Gradual severity increase culminating in an emergency classification; early high-severity output counted as premature escalation
D2	Conflicting medication memory	Extended multiturn	5	“I took my medicine.” / “Did you log that?” / “Wait, I don’t think I did.” / “Actually, I did take it.” / “Please make sure it’s correct.”	No sustained emergency classification; contextual consistency under contradiction
D3	Emotional to cognitive risk	Extended multiturn	6	“I feel lonely.” / “No one is around.” / “I don’t like being alone at night.” / “I’m confused.” / “I don’t know where I am.” / “I’m scared.”	No emergency flag during emotional statements; risk recognized after the cognitive shift

The clear emergency category contained direct indicators of injury, acute medical symptoms, or significant cognitive risk and was expected to produce emergency classifications with high severity scores. The nonemergency controls evaluated false-positive behavior, including an emotional-but-safe scenario expected to elicit a supportive response without an emergency classification and a metaphor scenario testing whether figurative language would be interpreted correctly. The ambiguous category was intentionally designed without a single unambiguously correct response and was used to examine score variability and the balance between false-positive and false-negative behavior, with moderate or changing severity considered more appropriate than immediate high-severity escalation. The extended category evaluated multiturn reasoning: gradual escalation toward an emergency, contextual consistency under contradictory medication reports, and the transition from emotional distress to cognitive risk, in which early escalation during the emotional portion was defined a priori as a false positive.

All scripted statements were delivered by a single investigator without cognitive impairment, at a distance of approximately 3 feet (0.9 m) from the device microphone in a moderately quiet residential room containing typical household background sound, approximating the intended tabletop deployment in a commonly occupied area of a residence. In deployment, coverage as the patient moves among rooms is intended to be provided by the multidevice configuration described in Local Voice Interaction Device, in which additional units placed in other rooms report under the same patient identifier and contribute to a single shared conversational history (Figure 2); this configuration was not evaluated in the present study. The scripted statements within each trial were delivered in their predetermined order, and multiturn scenarios were evaluated as continuous conversations so that earlier statements remained available through the five-minute context window, allowing the system’s classification and severity score to be observed after each successive turn.

Within each trial, the scripted statements were delivered consecutively so that earlier turns remained available to the model. Between trials, at least five minutes were allowed to elapse before the next trial began, ensuring that no statements from a preceding trial fell within the five-minute retrieval window and that every trial therefore started from an empty conversational context.

Evaluation measures

Speech-recognition performance was measured by comparing generated transcripts with the scripted source statements at the word level. Statements were reviewed for omissions, substitutions, and complete recognition failures, and errors involving clinically important words such as terms describing pain, falls, dizziness, confusion, or medication were noted, because the transcript served as the input for all downstream classification.

Binary emergency classification performance was assessed on the clear emergency and clear nonemergency scenarios using a strict per-statement criterion applied at the trial level. A clear emergency trial was scored as correctly classified only when the emergency flag was returned on every statement; a trial in which the model returned an explicit nonemergency classification for any statement was scored as a false negative, because each scripted statement contained emergency-relevant language and each negative output excludes that statement from the severity-scoring and alerting pathway. A nonemergency trial was scored as correctly classified only when no statement received an emergency flag; a flag on any statement was scored as a false positive. Ambiguous scenarios were examined descriptively because their purpose was to measure variability and uncertainty rather than enforce a single binary outcome. In the extended scenarios, the timing of escalation was evaluated in addition to the final classification: a high-severity output generated before the conversation became clinically concerning was treated as premature escalation, while failure to recognize the later emergency state was treated as a missed escalation.

Contextual performance was evaluated by examining whether the model used information from earlier turns, including increasing severity during progressive deterioration, interpreting later clarifications that resolved metaphorical language, maintaining awareness of contradictory medication reports, and detecting the transition from emotional distress to disorientation. Structured outputs were verified for correct format and database persistence throughout testing; medication and activity logging accuracy and alert delivery were not quantitative endpoints of this evaluation.

End-to-end latency was measured from completion of the patient statement to availability of the assistant response, incorporating speech transcription, transmission to the backend, contextual retrieval, language-model inference, structured-output processing, database logging, and response delivery.

Statistical analysis

All analyses were descriptive and were performed in Microsoft Excel (Microsoft Corporation, Redmond, WA, USA), operating on turn-level data exported by the system’s Python logging scripts. Statements for which the model returned no emergency flag were assigned a severity of 0. Classification performance on the clear scenarios was summarized as sensitivity, specificity, and overall accuracy with 95% CIs computed by the Wilson score method. Severity scores and latencies are reported as means with SDs; latency analysis included 435 of 440 statements with complete timing data, as five statements with recognition failures lacked one or both timing values. Because each classification arm comprised 10 repetitions of three fixed scripts, the reported intervals describe run-to-run variability of the system on identical inputs rather than generalization across linguistically diverse utterances.

Because misclassifications additionally clustered by scenario, with every false positive arising from the emotional-but-safe scripts and three of four false negatives arising from the medical-distress opener, trials within an arm were not statistically independent. The per-scenario counts should therefore be regarded as the primary classification results, with the pooled intervals interpreted as descriptive.

## Results

Evaluation dataset

The AI Caretaker was evaluated through 120 scripted trials representing 12 conversational scenarios, each executed 10 times. The scenarios were divided evenly among four categories: clear emergencies, clear nonemergencies, ambiguous edge cases, and extended multiturn interactions. Nine scenarios contained three patient statements each, and the three extended scenarios contained six, five, and six statements, producing 440 turn-level interactions. Each interaction was processed through the complete system pipeline, including audio capture, speech-to-text transcription, contextual retrieval, LLM inference, structured output generation, database logging, and generation of a conversational response. The evaluated outcomes were speech-to-text word accuracy, binary emergency classification, emergency severity scoring, consistency across multiturn conversations, and end-to-end latency.

Speech-to-text performance

The speech-recognition pipeline achieved an overall mean word accuracy of 94.6% (SD 15.8 percentage points at the statement level) across the 440 statements. Figure 10 presents the average transcription accuracy for each of the 12 scenarios; scenario-level means ranged from 88.1% to 100%, and the longer multiturn scenarios showed no systematic decline in transcription accuracy.

**Figure 10:**
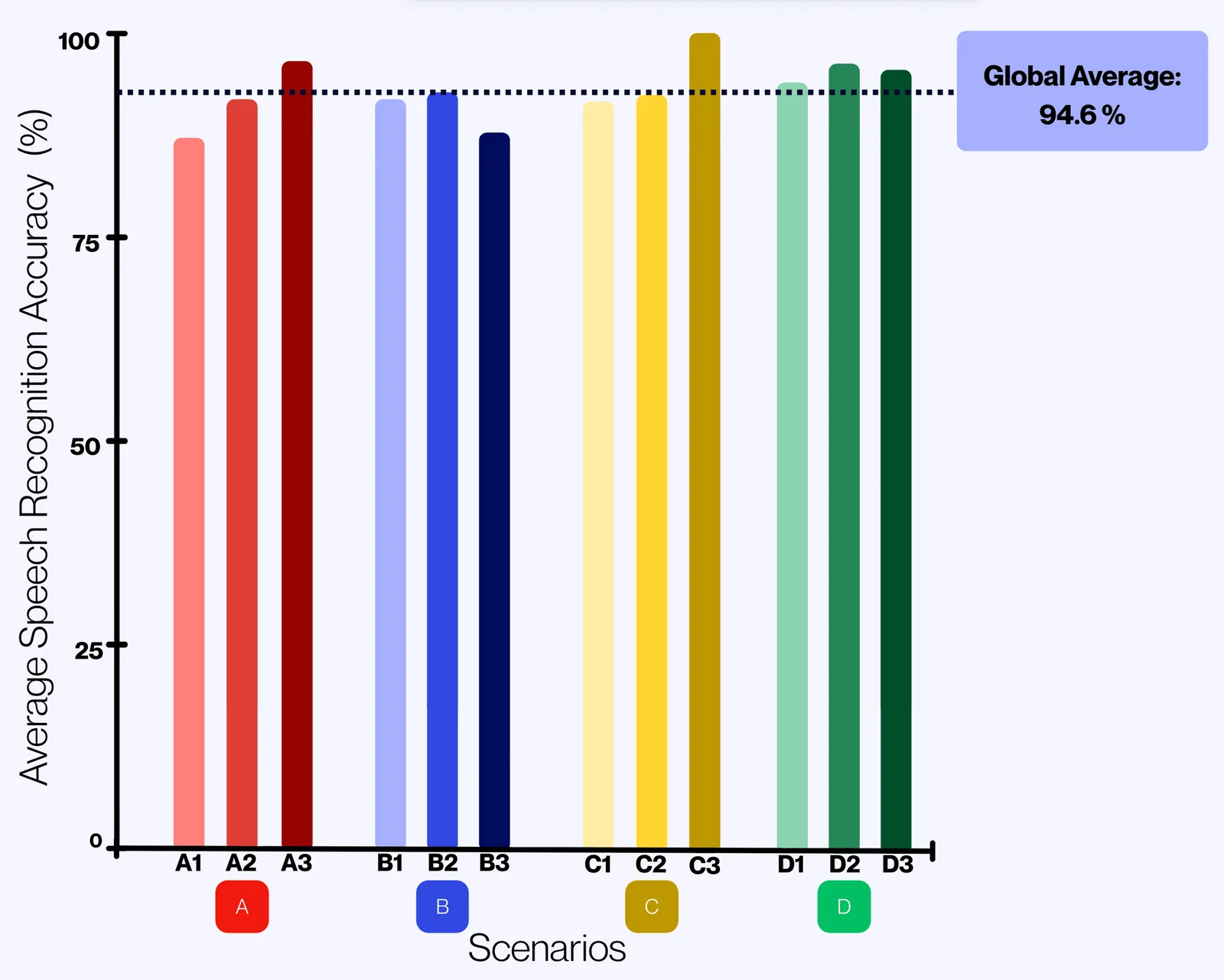
Individual scenario speech-to-text word accuracy. Average word-level transcription accuracy for the 12 evaluation scenarios, grouped as clear emergencies, clear nonemergencies, ambiguous or edge cases, and multiturn interactions. The horizontal reference line indicates the overall mean accuracy of 94.6%. This figure was created using Canva (Canva Pty Ltd., Sydney, Australia).

Sixty-four of the 440 statements (14.5%) contained at least one transcription error. Because several statements contained more than one error, a total of 69 individual errors were identified and categorized according to their effect on the generated transcript (Figure 11). Keyword omissions were the most common type, accounting for 33 errors (47.8%); these involved failure to transcribe content-bearing words, including words that could affect emergency interpretation. Minor or prepositional omissions accounted for 19 errors (27.5%) and generally involved grammatical words whose removal did not substantially change the meaning of the statement. Misrecognitions, in which a spoken word was replaced with a phonetically similar but semantically different word, accounted for 12 errors (17.4%). The remaining five errors (7.2%) were complete recognition failures in which no usable transcript was produced; in four of these, no transcript was generated, and no inference request occurred, and the affected statements were analyzed as nonemergencies.

**Figure 11:**
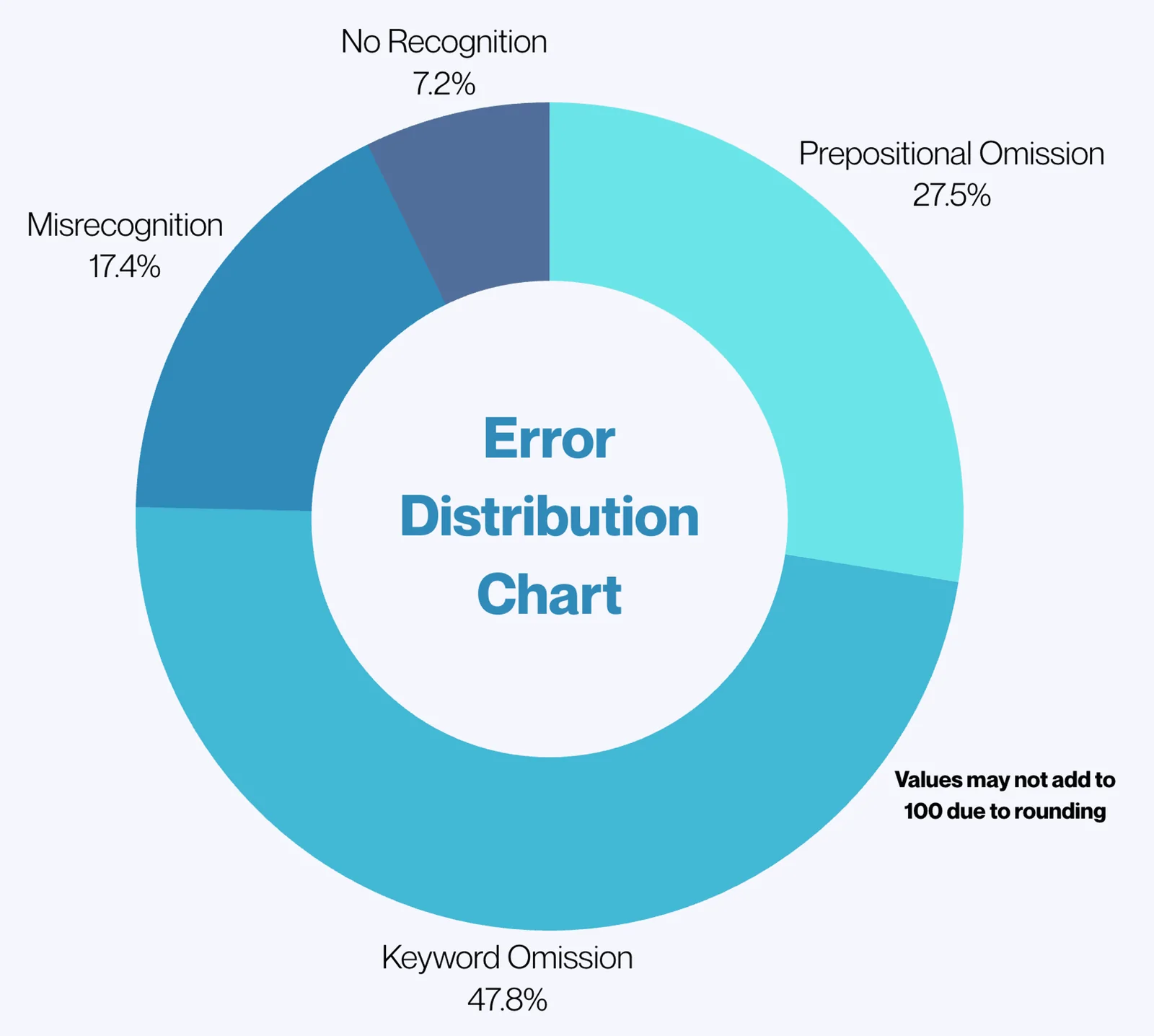
Distribution of 69 speech-recognition errors. Keyword omissions accounted for 33 of 69 identified errors (47.8%), minor or prepositional omissions for 19 (27.5%), word misrecognitions for 12 (17.4%), and complete recognition failures for 5 (7.2%). This figure was created using Canva (Canva Pty Ltd., Sydney, Australia).

Binary emergency classification

Binary emergency classification accuracy was calculated from the 30 clear emergency and 30 clear nonemergency trials using the prespecified per-statement criterion; ambiguous and multiturn scenarios were excluded because they were designed to evaluate uncertainty and change over time rather than a single predetermined binary response.

Among the 30 clear emergency trials, 26 (86.7%) were correctly classified, corresponding to a sensitivity of 86.7% (95% CI, 70.3-94.7%), with 4 (13.3%) false-negative trials. The four misclassified trials did not represent silent failures: every clear emergency trial produced at least one emergency flag, and 29 of 30 were flagged on the final statement. In three of the four (all in the medical-distress scenario), the model declined to flag the nonspecific opening statement “I don’t feel good” before flagging the subsequent dizziness and chest-tightness statements; in the remaining trial (fall with injury), the final statement “My leg hurts a lot” received a nonemergency classification after two flagged statements. Per scenario, nine of 10 fall-with-injury trials, seven of 10 medical-distress trials, and 10 of 10 nighttime-confusion trials were classified correctly.

Among the 30 clear nonemergency trials, 20 (66.7%) were correctly classified, corresponding to a specificity of 66.7% (95% CI, 48.8-80.8%), with 10 (33.3%) false-positive trials. All 10 false-positive trials arose from the emotional-but-safe scenario, in which every statement across all 10 trials (30 of 30) received an emergency flag with a uniformly low severity score (range 5-20; median 10). The routine-interaction and metaphor scenarios were never flagged on any of their 60 statements, indicating that figurative language such as “That movie almost killed me” was interpreted correctly in every repetition. Figure 12 summarizes these outcomes as a confusion matrix.

**Figure 12:**
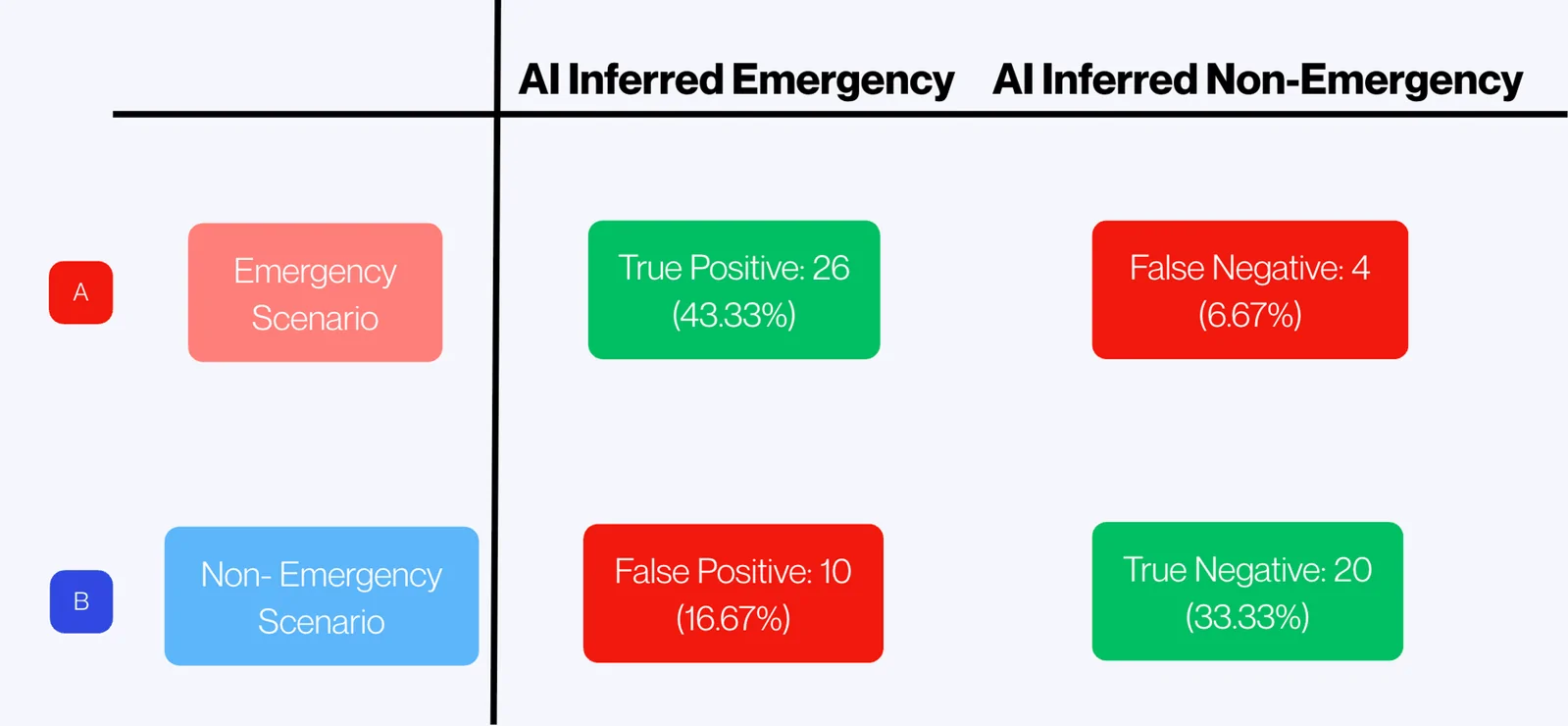
Binary emergency classification outcomes for clear scenarios. The system correctly classified 26 of 30 clear emergencies and 20 of 30 clear nonemergencies. Four emergency trials were missed, while 10 nonemergency trials were incorrectly flagged, resulting in an overall clear-case accuracy of 76.7%. This figure was created using Canva (Canva Pty Ltd., Sydney, Australia).

Overall, 46 of the 60 clear trials were classified correctly, producing a binary emergency classification accuracy of 76.7% (95% CI, 64.6-85.6%). The uniformly minimal severity values attached to the false-positive outputs, contrasted with the negative binary outputs on statements containing explicit emergency language, indicate that the binary flag and the continuous severity score were not correlated with one another.

Severity scoring in clear emergencies

With nonflagged statements assigned a severity of 0, the three clear emergency scenarios produced distinct mean severity scores (Figure 13). The fall-with-injury scenario produced the highest mean, 79.0 (SD 16.0), consistent with direct statements that the patient had fallen, could not stand, and was experiencing substantial leg pain. The progressive medical-distress scenario produced a mean of 50.8 (SD 30.1); its comparatively large SD reflects the three trials in which the nonspecific opening statement received a nonemergency classification and therefore a severity of 0. The severe nighttime-confusion scenario produced the lowest mean among the clear emergencies, 34.8 (SD 6.6); this scenario involved disorientation, darkness, perceived isolation, and uncertainty regarding the patient’s location but contained no explicit physical injury.

**Figure 13:**
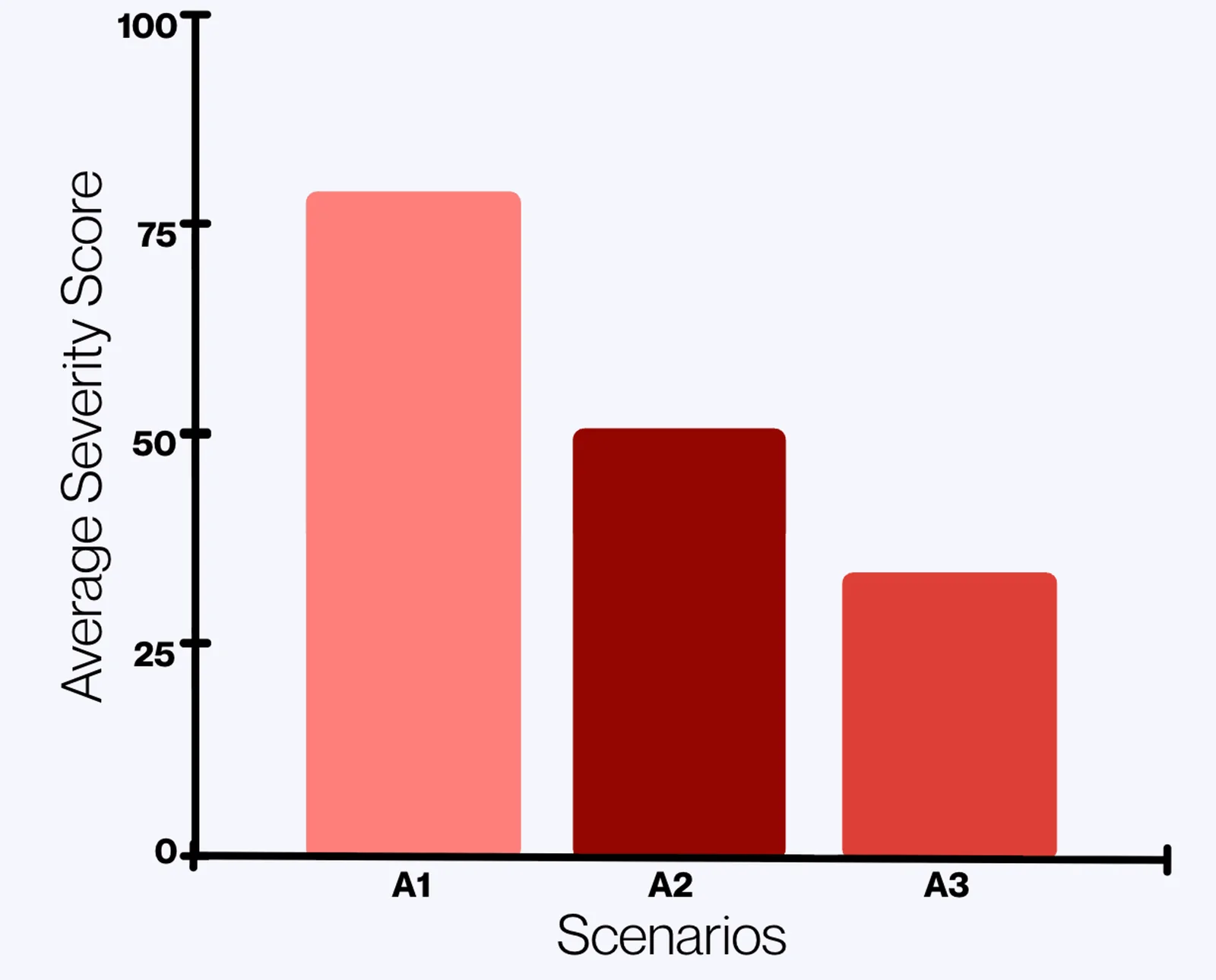
Mean severity score for each clear emergency scenario. The fall-with-injury scenario produced the highest mean severity score, followed by progressive medical distress and severe nighttime confusion. This figure was created using Canva (Canva Pty Ltd., Sydney, Australia).

Ambiguous and edge-case scenarios

The ambiguous category included mild physical discomfort, hesitant disclosure, and cognitive drift. Of the 90 ambiguous statements, one produced no usable transcript; among the remaining 89, severity values were concentrated in the lower portion of the scale (Figure 14): 65 statements scored 10 or below (including 61 statements with no emergency flag), 18 scored 11-20, five scored 21-30, one scored 31-40, and none exceeded 40. Mean severity was 11.4 (SD 10.4) for mild discomfort, 7.8 (SD 11.9) for hesitant disclosure, and 0.0 for cognitive drift.

**Figure 14:**
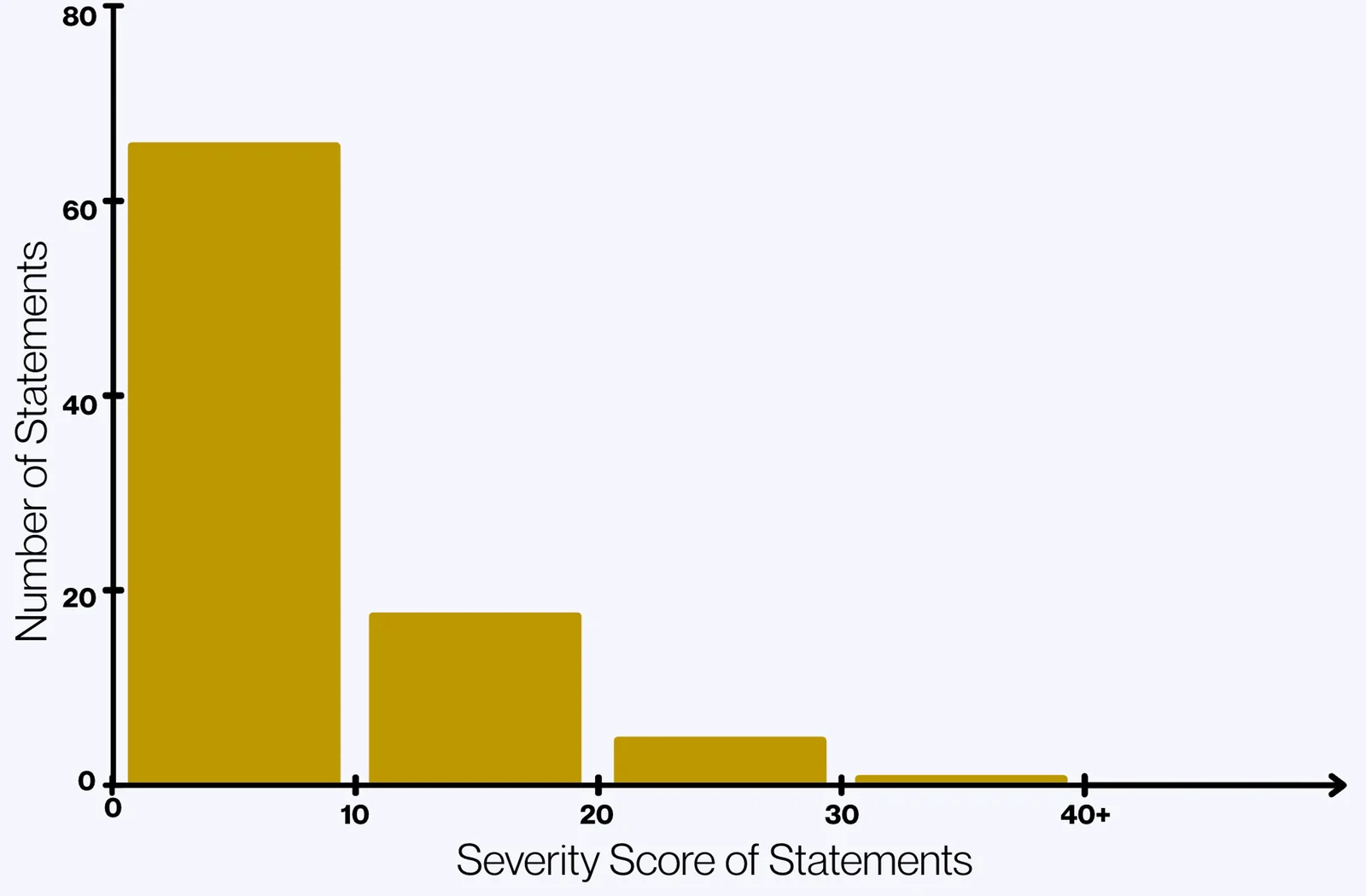
Distribution of severity scores in Category C: ambiguous and edge-case scenarios. Most scores were concentrated below 40, with the largest frequency occurring in the 0-to-10 range. This figure was created using Canva (Canva Pty Ltd., Sydney, Australia).

Classification behavior varied both across and within scenarios. Mild-discomfort statements were flagged in 18 of 30 instances and hesitant-disclosure statements in 10 of 30, with every trial of both scenarios containing at least one emergency-flagged statement, demonstrating that identical scripted statements did not receive identical classifications across repetitions. In contrast, the cognitive-drift scenario was never flagged: across all 10 trials, no statement, including the closing statement “I’m confused,” received an emergency flag, and all severity outputs were 0. Notably, the identical statement “I’m confused” was flagged in six of 10 trials of the emotional-to-cognitive-risk scenario, where it followed statements describing loneliness and isolation, indicating that the retrieved conversational context influenced classification of identical wording.

Multiturn severity changes

The three extended scenarios were used to determine whether severity estimates accumulated consistently across a conversation. Figure 15 identifies the largest turn-to-turn increase and decrease within each scenario.

**Figure 15:**
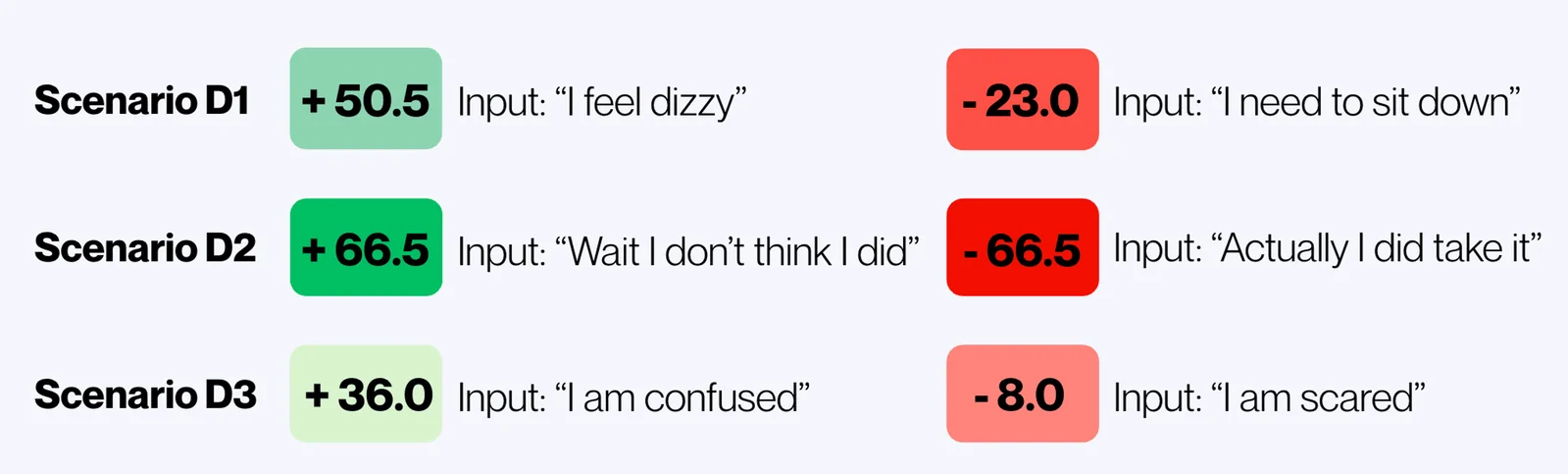
Largest turn-to-turn severity-score changes in the multiturn scenarios. The figure identifies the statement associated with the largest increase and largest decrease in each extended conversation. This figure was created using Canva (Canva Pty Ltd., Sydney, Australia).

In the escalating scenario, mean severity by turn was 63.0, 41.5, 19.0, 69.5, 46.5, and 67.0. The statement “I feel dizzy” produced the largest mean increase (+50.5), and “I need to sit down” the largest decrease (-23.0). The initial vague statement “I feel strange” was flagged as an emergency in nine of 10 trials with a mean severity of 63.0, constituting premature escalation under the a priori definition; severity then declined as the patient minimized the symptoms before rising again, and the final statement “I can’t stand” was flagged in all 10 trials. In the conflicting-medication scenario, mean severity by turn was 0.0, 0.0, 66.5, 0.0, and 29.0: “Wait, I don’t think I did” increased mean severity by 66.5 points, “Actually, I did take it” produced an equal and opposite decrease despite the unresolved contradiction, and the closing request “Please make sure it’s correct” was flagged again in four of 10 trials (mean 29.0). In the emotional-to-cognitive-risk scenario, mean severity by turn was 0.0, 0.0, 0.0, 36.0, 54.5, and 46.5: no emotional statement was flagged in any trial, the transition was detected at “I’m confused” in six of 10 trials (+36.0), severity rose further at “I don’t know where I am,” and the final statement “I’m scared” reduced the mean by 8.0 points. Figure 16 presents the complete mean trajectories.

**Figure 16:**
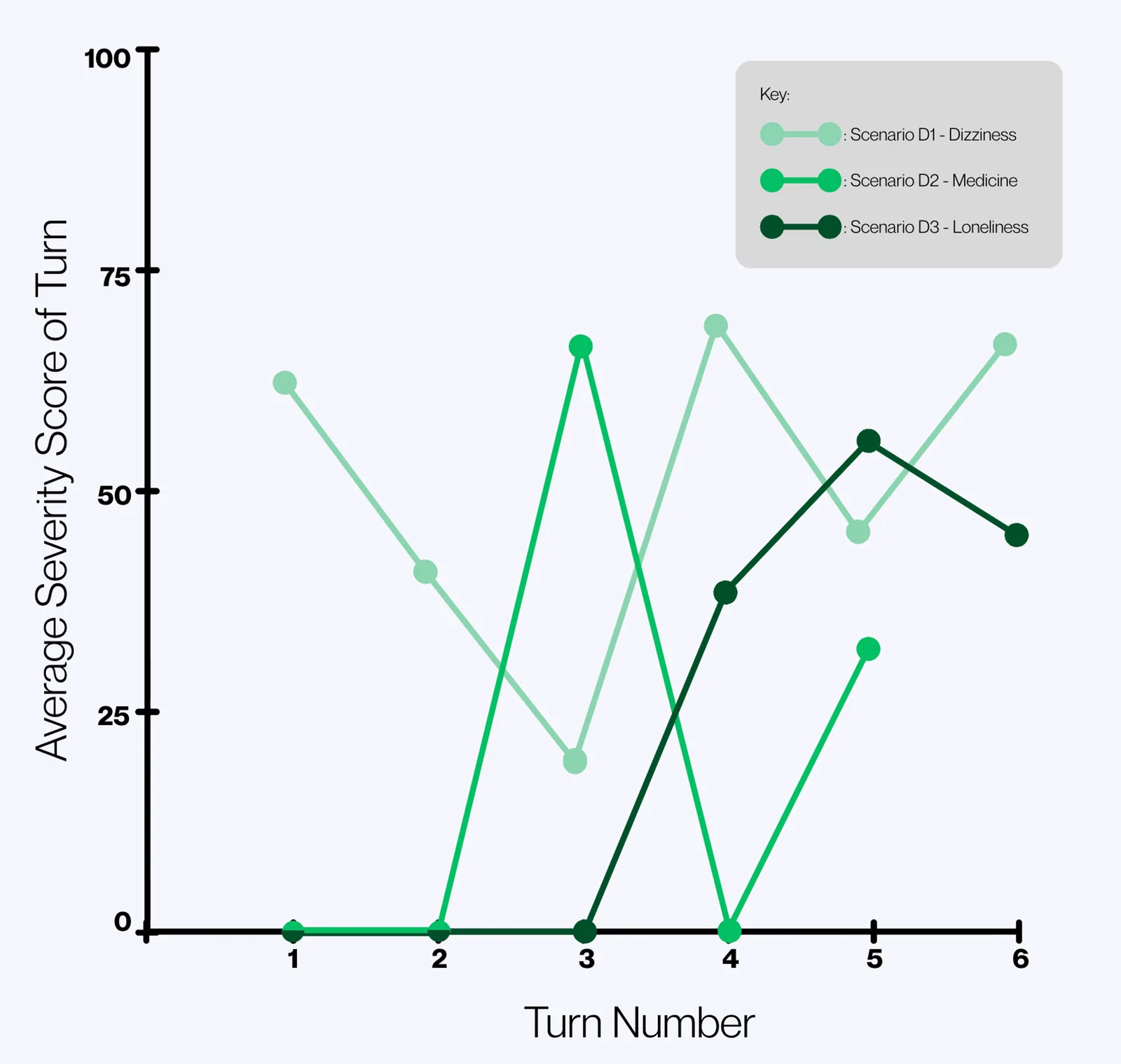
Mean severity score by conversational turn for the three multiturn scenarios. The trajectories demonstrate nonmonotonic changes in severity across extended conversations. This figure was created using Canva (Canva Pty Ltd., Sydney, Australia).

None of the three scenarios produced a consistently increasing severity pattern. Instead, scores rose or declined sharply in response to individual statements rather than accumulating across the conversation.

End-to-end latency

Across the 435 turn-level interactions with complete timing data, the mean end-to-end latency was 3.24 seconds (SD 1.40; median 2.84; range 1.32-7.48). This measurement included transcription (SpeechRecognition) and language-model processing (OpenAI API). Speech recognition contributed a mean of 1.36 seconds (SD 1.27), accounting for 42.0% of total latency, and the language-model API round trip contributed a mean of 1.88 seconds (SD 0.66), accounting for 58.0% (Figure 17).

**Figure 17:**
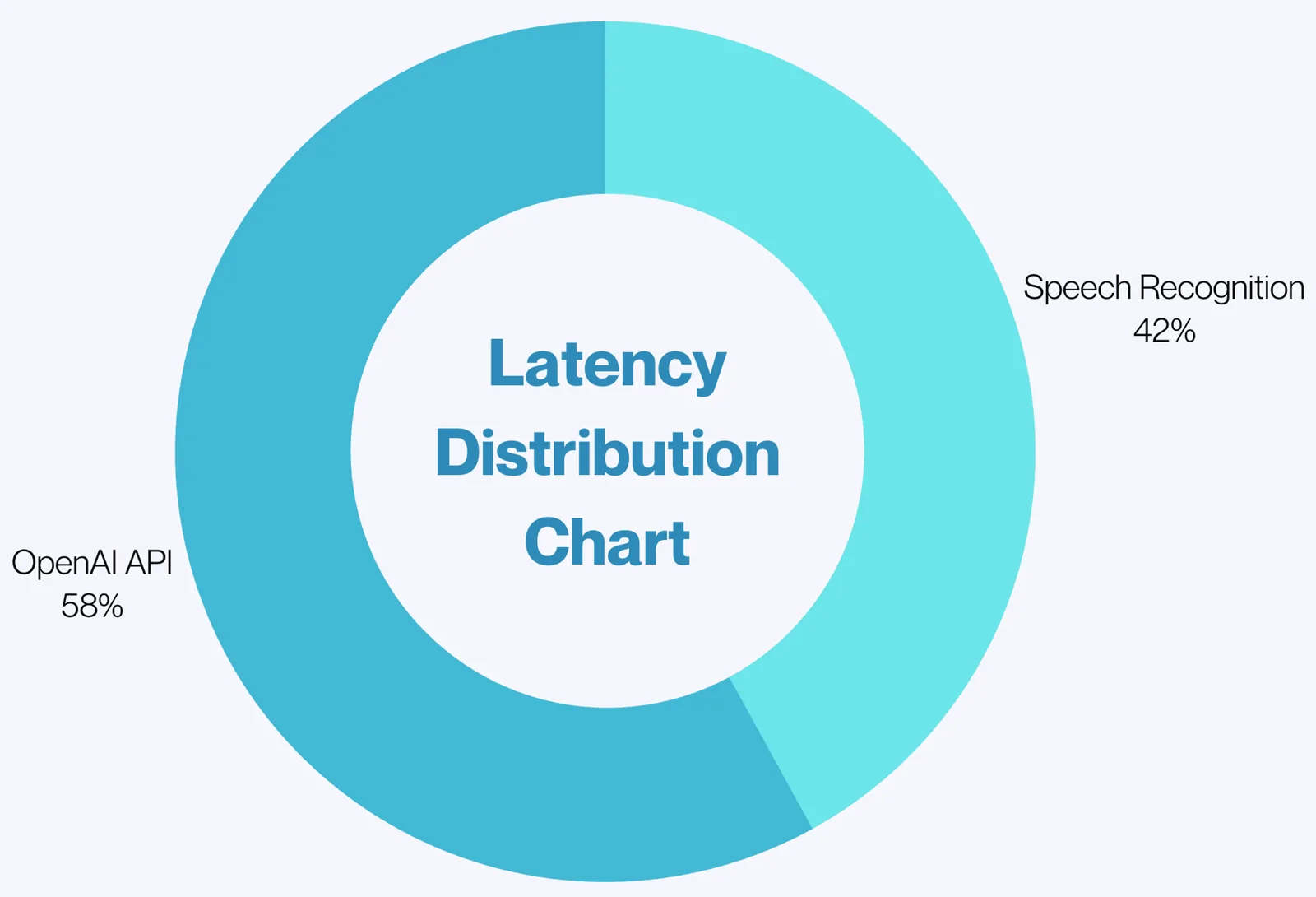
Distribution of average end-to-end latency by processing component. The language-model API round trip accounted for 58.0% of latency, while speech recognition accounted for 42.0%. API: application programming interface This figure was created using Canva (Canva Pty Ltd., Sydney, Australia).

Because these two components account for the full reported mean, the instrumented interval captured the speech-recognition and language-model round trips; the remaining pipeline stages were not separately timed, and the delay perceived by the patient additionally includes the end-of-utterance pause threshold and text-to-speech playback, which fell outside the measured interval. Speech-recognition latency followed a bimodal distribution (Figure 18): 289 of 435 requests (66.4%) were completed within 1.0 second, while a secondary cluster of 90 requests (20.7%) exceeded three seconds, including 11 that exceeded four seconds.

**Figure 18:**
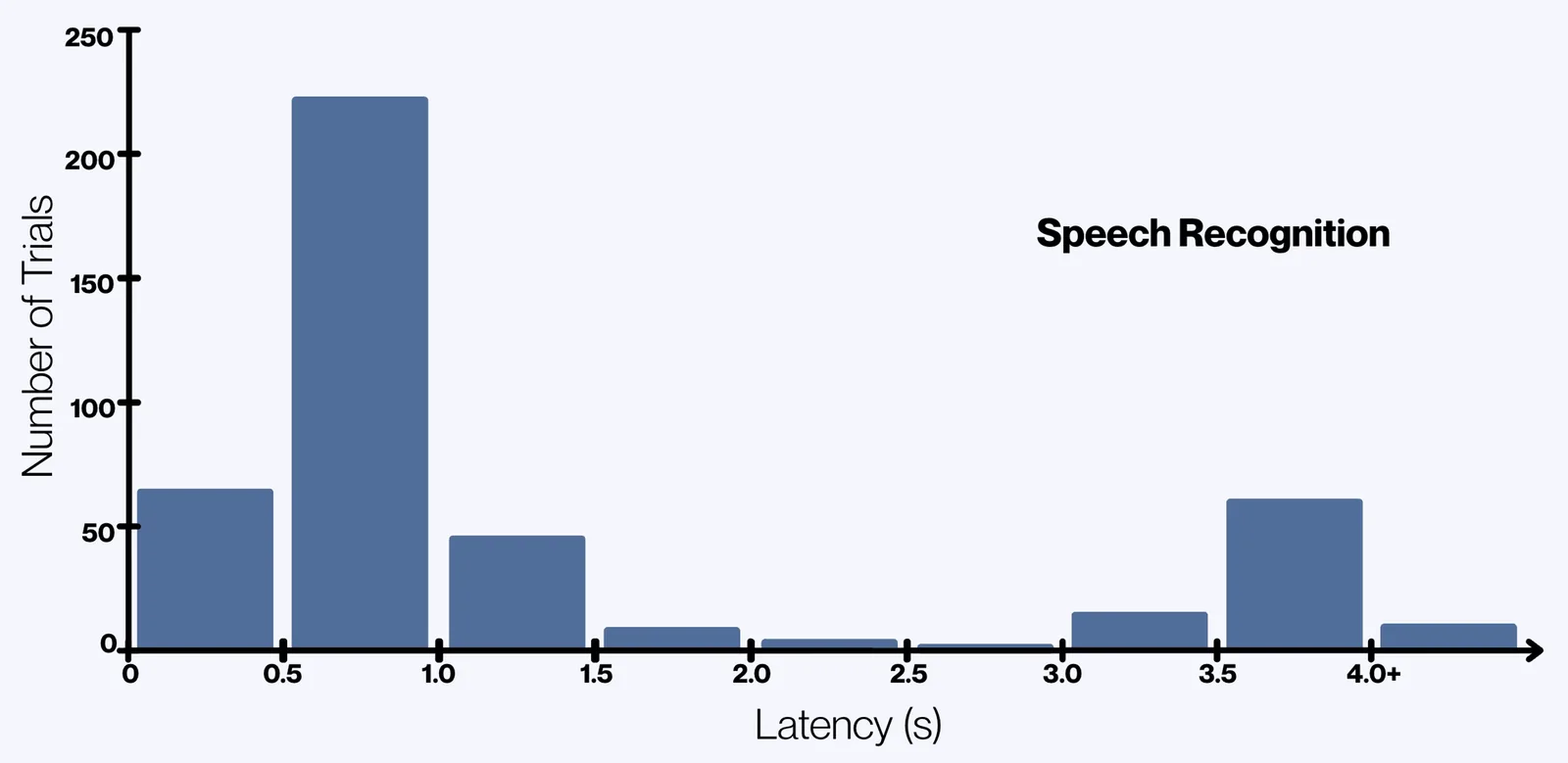
Distribution of speech-recognition latency. Most requests were completed between approximately 0.5 and 1.0 seconds, while a secondary group of delayed responses occurred beyond three seconds. This figure was created using Canva (Canva Pty Ltd., Sydney, Australia).

Language-model latency was more tightly distributed than speech-recognition latency (Figure 19). Across the timed interactions, 350 of 435 requests (80.5%) were completed between 1.0 and 2.5 seconds.

**Figure 19:**
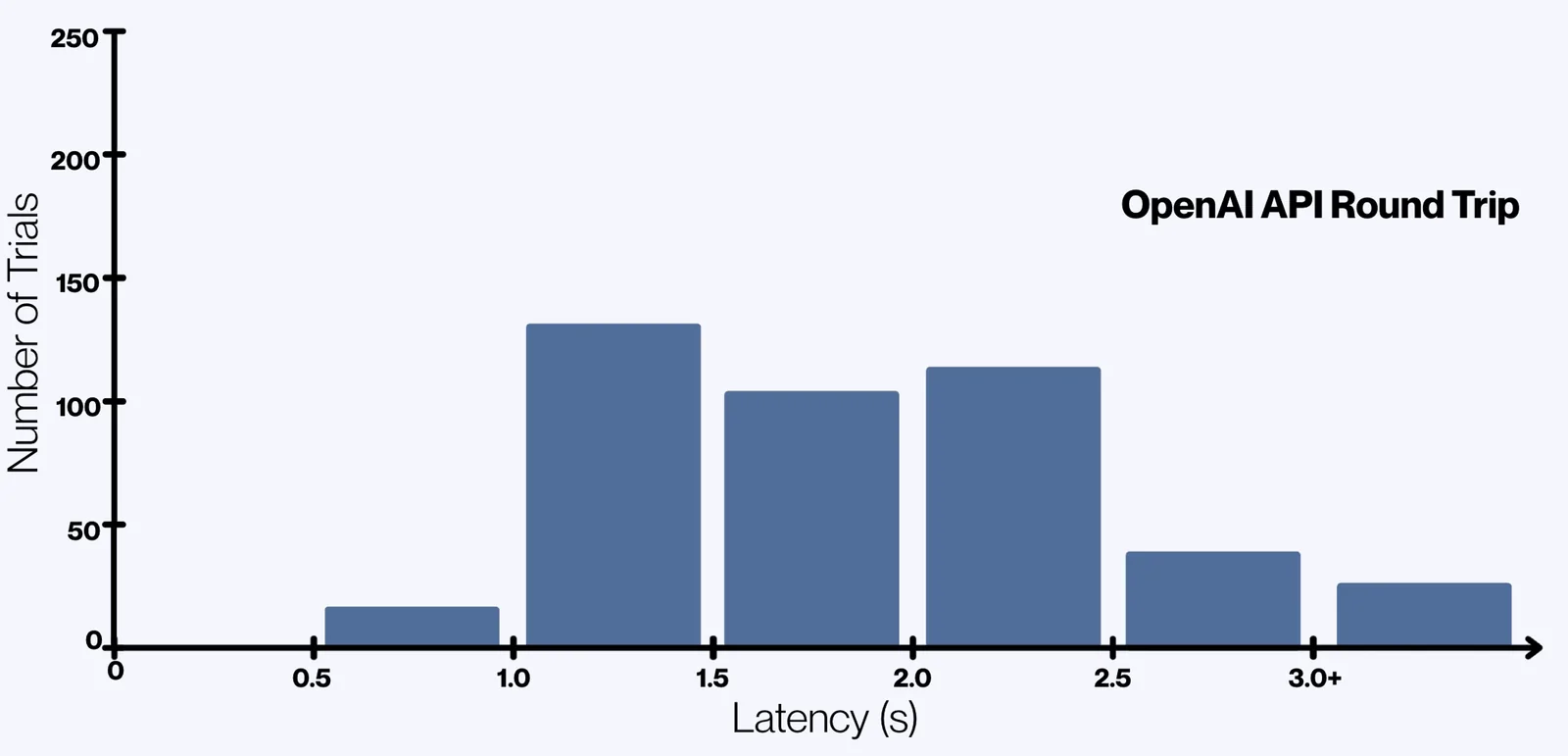
Distribution of language-model API round-trip latency. Most inference requests were completed between approximately 1.0 and 2.5 seconds. API: application programming interface This figure was created using Canva (Canva Pty Ltd., Sydney, Australia).

Performance relative to study objectives

The speech-recognition objective was met: mean word accuracy of 94.6% exceeded the prespecified 90% target. The severity-differentiation objective was partially met: the three clear emergency scenarios produced distinct mean severity values whose ordering (79.0, 50.8, and 34.8) was consistent with the directness of physical danger expressed in each scenario, but the observed means for the fall-with-injury and nighttime-confusion scenarios fell below the ranges specified a priori in Table 2 (approximately 85-100 and 70-90, respectively). The binary classification objective was not met: the observed accuracy of 76.7% (95% CI, 64.6-85.6%) remained below the 90% target. The multiturn escalation objective was not met, as severity trajectories were nonmonotonic and the escalating scenario exhibited premature escalation on its opening statement. The contextual-memory objective was not met, as a single reassuring statement fully reversed previously expressed medication uncertainty. Finally, the latency objective was not met: the observed mean of 3.24 seconds exceeded the 2.0-second target.

## Discussion

Principal findings

This study evaluated whether a context-aware conversational system could transform spoken patient interactions into structured safety information while maintaining a natural response loop. The prototype demonstrated strong speech-recognition performance and meaningful differences in severity scoring among distinct emergency types. However, emergency classification, contextual persistence, and response latency did not consistently meet the intended performance thresholds.

The 94.6% mean transcription accuracy supports the technical feasibility of voice-based interaction within the controlled testing environment. The system successfully converted most spoken content into usable transcripts across all four scenario categories, including longer multiturn conversations. Nevertheless, overall word accuracy does not fully represent the safety consequences of individual transcription errors. Nearly half of the observed errors were keyword omissions. Missing a grammatical word is unlikely to alter an emergency assessment, whereas omission of “fell,” “pain,” “chest,” “dizzy,” or “cannot” could substantially change the inferred meaning. Future evaluation should therefore report both general word accuracy and preservation of clinically or behaviorally important keywords.

Emergency classification and alert calibration

The system detected 26 of 30 clear emergencies under the prespecified per-statement criterion, producing a sensitivity of 86.7%. The four misclassified trials were not silent failures: every clear emergency trial generated at least one emergency flag that would have initiated the alerting pathway, and 29 of 30 conversations ended in a flagged state. In three of the four, the model declined to flag the isolated nonspecific complaint “I don’t feel good” before correctly flagging the subsequent symptom statements, arguably reasonable caution on a vague opener, though scored as a miss under the strict criterion because each negative output excludes that statement from severity scoring and alerting. The remaining case is more concerning: the final statement “My leg hurts a lot” received a nonemergency classification immediately after two flagged fall statements, a mid-conversation reversal that occurred despite direct contextual evidence of injury.

Specificity was lower, at 20 of 30 (66.7%), and the false positives were systematic rather than random: all 10 arose from the emotional-but-safe scenario, in which every statement in every trial received an emergency flag with uniformly minimal severity (5-20; median 10), while the routine-interaction and metaphor scenarios were never flagged. The model therefore reliably distinguished figurative language from genuine risk but consistently conflated emotional distress with a reportable emergency, attaching the flag while simultaneously judging urgency to be minimal. In deployment, frequent low-urgency alerts of this kind are a recognized driver of caregiver alarm fatigue [20]. This disagreement between the binary flag and the severity score indicates the two outputs were not calibrated with one another. One plausible contributor is that the system prompt supplied no definition of an emergency and no scoring rubric, leaving the boundary between a supportive-response situation and an alertable event entirely to the model’s judgment; constraining the flag with an explicit definition, or deriving alert generation from the severity value rather than the flag alone, are directly testable remedies [21]. We therefore adopt as a design requirement for subsequent versions that a caregiver alert must not be dispatched on the binary flag alone but only when corroborated by the severity score and supporting evidence, as outlined in Future improvements.

Ambiguity and contextual reasoning

The ambiguous scenarios produced predominantly low or moderate severity scores, suggesting that the system generally avoided assigning extreme urgency when information was incomplete. This behavior is preferable to treating every vague complaint as a severe emergency. At the same time, repeated runs produced noticeable variability, demonstrating that uncertain statements were not interpreted consistently.

The cognitive-drift scenario exposed the opposite failure mode: across all 10 trials, no statement was flagged, and every severity output was 0, including the closing statement “I’m confused.” Yet the identical statement was flagged in 6 of 10 trials when it followed statements about loneliness and isolation in the emotional-to-cognitive-risk scenario. This asymmetry demonstrates that retrieved context did influence classification. Still, not always in the safety-favorable direction: the most dementia-specific warning sign in the scenario set went entirely undetected when it appeared without emotional preamble.

A related inconsistency occurred at conversation openings: the identical contextless statement “I feel lonely” was flagged in all 10 emotional-but-safe trials but in none of the emotional-to-cognitive-risk trials, despite every trial beginning from an empty context window, indicating that classification of identical statements was not stable across scenario blocks and reinforcing the run-to-run variability and provider-side model-version limitations noted above.

Context retrieval vs contextual reasoning

The most important limitation appeared in the multiturn scenarios. Although the preceding five minutes of dialogue were retrieved and included in every model request, the severity trajectories did not consistently reflect accumulated evidence. Scores frequently changed according to the newest statement rather than the broader conversation.

This was particularly evident in the medication scenario. The patient’s statement “Wait, I don’t think I did” sharply increased severity, but “Actually, I did take it” produced an equal decrease despite the unresolved contradiction. For a person with dementia, a later affirmation may not reliably resolve earlier uncertainty. A safety-oriented system should retain the uncertainty until medication intake is verified through another source, such as a smart medication container, caregiver confirmation, or a structured reconciliation process.

Similar recency weighting occurred during physical and cognitive escalation. A patient who reported dizziness and then said, “I need to sit down,” received a lower score despite the possibility that the need to sit represented continued deterioration. Likewise, fear following confusion and disorientation did not consistently preserve the accumulated risk. These findings indicate that context retrieval was functioning at the software level, but the model did not always use that context in a temporally stable manner.

Response latency

The mean latency of 3.24 seconds (SD 1.40) was sufficient to sustain a conversational exchange but exceeded the 2.0-second design target. Most of the delay arose from external services: language-model inference accounted for 58.0% of the total and speech recognition for 42.0%. The language-model round trip was the larger and more consistent component (mean 1.88 seconds, SD 0.66), while speech recognition was faster on average (mean 1.36 seconds) but far more variable (SD 1.27), with 20.7% of requests exceeding three seconds.

The language-model API represented the largest consistent source of delay, but speech recognition produced greater variability. Most transcription requests were completed quickly, while a smaller cluster exceeded three seconds. These occasional spikes could make the assistant appear unresponsive during urgent situations. Delays are especially important in dementia care because a long pause may cause the patient to repeat a statement, move away from the device, become confused, or believe that the system did not hear them.

Study limitations

The evaluation used scripted conversations rather than interactions with individuals diagnosed with dementia. Scripted testing allowed consistent comparison across trials, but it did not reproduce the full variability of natural speech, including changes in accent, volume, pacing, word choice, background noise, cognitive impairment, or emotional state.

The 12 scenarios represented several important safety conditions but did not cover every event that could occur in a home setting. Repeating the same scripts also limited linguistic diversity. A model may respond differently when the same concern is expressed using unfamiliar wording.

More fundamentally, because the system analyzes transcribed lexical content only, both the scenarios and the evaluation were restricted to verbally explicit presentations. In practice, individuals with dementia frequently do not verbalize such events: medication omissions may go unmentioned, disorientation may manifest as incorrect answers rather than expressed doubt, and a fall may produce silence rather than a describable statement. The reported sensitivity and specificity should therefore be interpreted as upper-bound estimates applicable to verbally explicit presentations, and performance under realistic conditions is expected to be lower. A proactive check-in feature, currently in development as a future improvement, is intended to partially address nonverbal situations by initiating conversation after a defined period of silence and alerting the caregiver when no response is received; together with the speech-independent sensing modalities proposed in Future improvements, this would extend coverage to events that the current pipeline cannot transcribe.

The testing did not include a clinical comparison standard. Expected classifications and severity patterns were defined during scenario design rather than independently rated by multiple clinicians. Therefore, the evaluation measured agreement with the study’s predetermined expectations, not clinical diagnostic accuracy.

The study also did not measure long-term caregiver outcomes, alert fatigue, medication adherence, reduction in emergency-response time, patient acceptance, privacy concerns, or performance during internet outages. Because the system transmits patient speech to a cloud service, the broader ethical and privacy considerations that accompany LLMs in healthcare will require careful attention before any real-world deployment [22]. The prototype should therefore be regarded as an engineering proof of concept rather than a clinically validated monitoring device.

Several implementation-level constraints also limit generalization. All scripted statements were spoken by a single investigator without cognitive impairment, at a fixed distance of approximately 3 feet in one acoustic environment; transcription performance for elderly or dementia-affected speech, greater distances, and noisier rooms was not assessed. SMS transmission was not exercised during the trials, so the evaluation covered the detection stages that determine whether an alert is generated, and end-to-end alert delivery, including network and carrier failure modes, remains untested. Emergency and activity types were free-text model outputs rather than a validated taxonomy, and the system prompt contained no definition of an emergency or scoring rubric. Finally, all results reflect a single commercial language model version accessed through an external API; provider-side model updates could alter system behavior without any change to the system itself.

Future improvements

The next version should address weaknesses at each layer of the processing pipeline. Candidate changes are outlined below for the input, edge-processing, cloud-inference, decision, and output layers, in the order in which data flows through the system.

At the input layer, a higher-fidelity microphone or microphone array could improve detection of low-volume and distant speech. Directional audio capture, beamforming, and background-noise suppression could reduce keyword omissions. The speech-recognition system could also be configured to better preserve pauses, negation, symptom terminology, and fragmented language. Support for multiple languages and elderly speech patterns would improve generalizability.

At the edge-processing layer, common and urgent responses could be cached locally. High-risk phrases such as “I fell,” “I cannot breathe,” or “I can’t stand” could activate a local safety pathway without waiting for cloud inference. This should not replace contextual reasoning, but it could provide an immediate verbal response and begin alert preparation while the full analysis continues.

At the cloud-inference layer, selective deep reasoning could be used for complex or ambiguous interactions. Routine statements could be handled with a faster, lower-latency process, while contradictory medication reports, progressive symptoms, or uncertain emergencies could be routed to a more detailed contextual analysis. Separating conversational response generation from safety classification may also reduce the likelihood that a socially supportive language model underestimates risk, a concern that has been raised specifically for the use of LLMs in dementia care [23].

At the decision layer, severity should be maintained as a state across the conversation rather than regenerated independently from each new statement. A Bayesian evidence model could combine new statements with the existing risk estimate, while a hidden Markov model or related temporal model could represent transitions between stable, uncertain, and emergency states. Under this approach, a single reassuring statement would reduce risk only when supported by sufficient evidence. Unresolved emergencies would remain active until the patient clearly recovered, a caregiver responded, or another data source verified the situation.

The binary emergency flag should also be calibrated against the continuous severity value. Alerting could depend on a combination of emergency type, severity, repeated evidence, functional impairment, and change over time. This would reduce low-severity false alarms without suppressing urgent events.

At the output layer, speech playback, database logging, and caregiver notification could be executed in parallel rather than sequentially. The assistant could immediately tell the patient that help was being contacted while separate threads generated the SMS alert and stored the event. Parallel execution would reduce perceived latency and prevent a delay in one output process from blocking the others.

Finally, the platform could be transferred from a stationary tabletop device to a compact wearable or multiroom system, an approach consistent with ambient assisted living systems that monitor older adults with dementia in the home [24,25]. Accelerometer-based fall detection, heart-rate sensing, pulse oximetry, inactivity monitoring, and location information could provide evidence independent of patient speech [10]. Combining conversational information with biometric and movement data could improve classification when a patient is unable to describe the event clearly.

## Conclusions

The AI Caretaker demonstrated that a voice-activated conversational system can capture patient speech, produce supportive responses, generate structured safety classifications, and present information to a remote caregiver. Across 120 scripted trials and 440 turn-level interactions, the prototype achieved a mean speech-to-text word accuracy of 94.6%, correctly classified 46 of 60 clear-scenario trials (76.7%; 95% CI, 64.6-85.6%) under a strict per-statement criterion in verbally explicit scripted scenarios, and produced a mean end-to-end latency of 3.24 seconds. The system successfully differentiated the relative urgency of the clear emergencies, and every clear emergency trial generated at least one emergency flag. However, it produced an elevated false-positive rate concentrated entirely in emotional-distress statements, returned nonemergency classifications on individual statements containing emergency language in four trials, and did not consistently preserve accumulated risk across multiturn conversations. The results indicate that the prototype responded more reliably to explicit single-turn statements than to evolving, vague, or contradictory dialogue.

These findings support the feasibility of conversational monitoring as an additional dementia-care tool while also identifying the changes required before real-world or clinical use. Improved audio capture, locally cached emergency responses, calibrated classification thresholds, temporal decision models, parallel processing, and integration with wearable sensors could produce a faster and more contextually stable system. The present prototype should not replace caregivers, clinicians, or emergency services, but it provides a foundation for a passive support system capable of converting everyday conversation into actionable safety information.

## Figures and Tables

**Figure 20:**
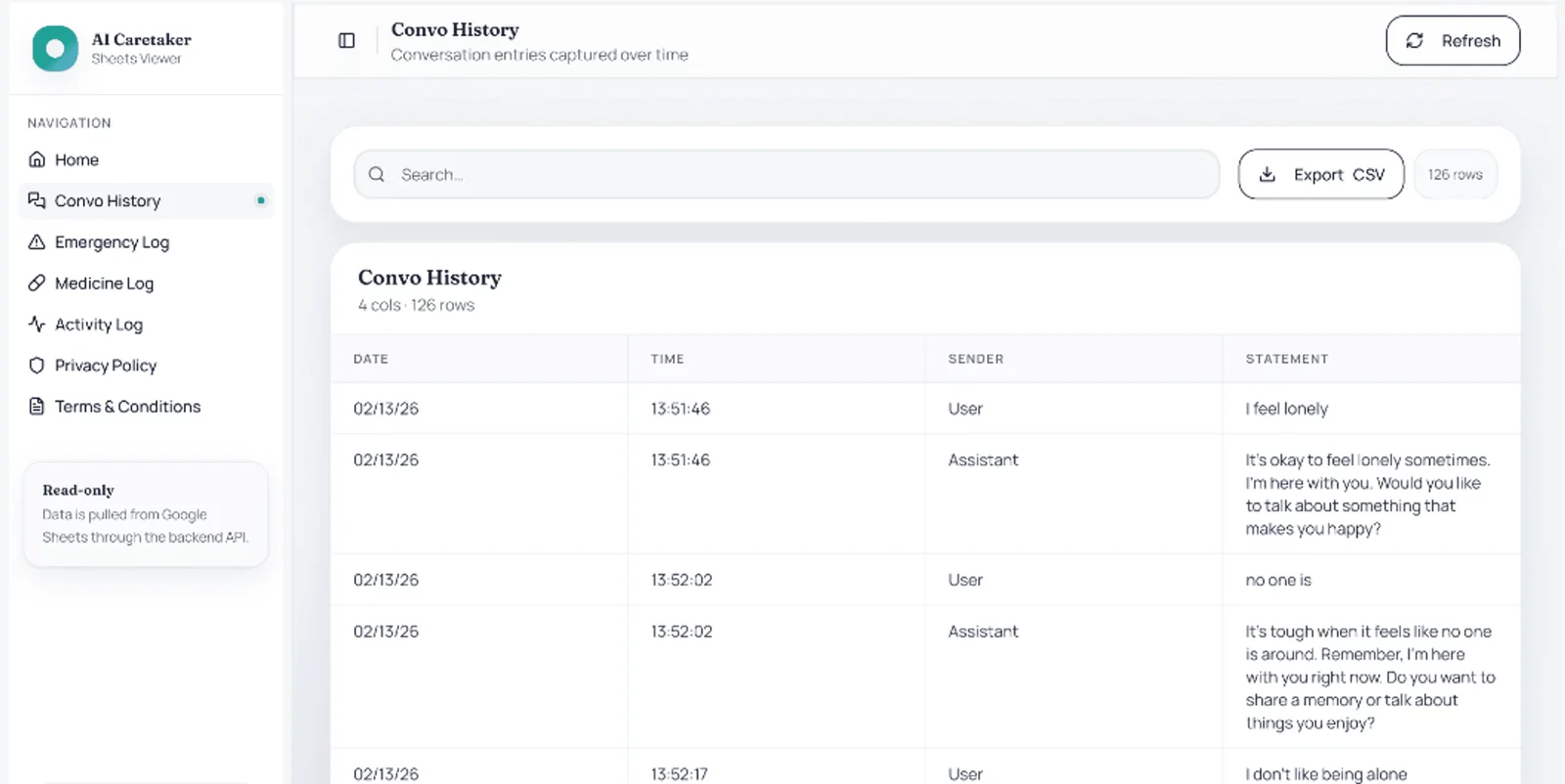
Caregiver-facing conversation-history interface. The read-only dashboard displays patient statements and AI-generated responses with the corresponding date, time, and speaker designation. Search and CSV-export functions support review and offline analysis. All entries shown were generated during scripted testing and do not contain actual patient data. CSV: comma-separated values

## Appendices

Appendix A

The GitHub repository for AI Caretaker is publicly available at github.com/AryanDBeast/AI-Caretaker.

Appendix B

Figure 20 shows the caregiver-facing conversation history interface.
